# Bloom syndrome DNA helicase deficiency is associated with oxidative stress and mitochondrial network changes

**DOI:** 10.1038/s41598-021-81075-0

**Published:** 2021-01-25

**Authors:** Veena Subramanian, Brian Rodemoyer, Vivek Shastri, Lene J. Rasmussen, Claus Desler, Kristina H. Schmidt

**Affiliations:** 1 Department of Cell Biology, Microbiology and Molecular Biology, University of South Florida, Tampa, FL 33620 USA; 2 Center for Healthy Aging, Department of Cellular and Molecular Medicine, University of Copenhagen, 2200 Copenhagen, Denmark; 3 Cancer Biology and Evolution Program, H. Lee Moffitt Cancer Center and Research Institute, Tampa, FL 33612 USA

**Keywords:** Cell biology, Mechanisms of disease, Cell division, DNA damage and repair, DNA replication

## Abstract

Bloom Syndrome (BS; OMIM #210900; ORPHA #125) is a rare genetic disorder that is associated with growth deficits, compromised immune system, insulin resistance, genome instability and extraordinary predisposition to cancer. Most efforts thus far have focused on understanding the role of the Bloom syndrome DNA helicase BLM as a recombination factor in maintaining genome stability and suppressing cancer. Here, we observed increased levels of reactive oxygen species (ROS) and DNA base damage in BLM-deficient cells, as well as oxidative-stress-dependent reduction in DNA replication speed. BLM-deficient cells exhibited increased mitochondrial mass, upregulation of mitochondrial transcription factor A (TFAM), higher ATP levels and increased respiratory reserve capacity. Cyclin B1, which acts in complex with cyclin-dependent kinase CDK1 to regulate mitotic entry and associated mitochondrial fission by phosphorylating mitochondrial fission protein Drp1, fails to be fully degraded in BLM-deficient cells and shows unscheduled expression in G1 phase cells. This failure to degrade cyclin B1 is accompanied by increased levels and persistent activation of Drp1 throughout mitosis and into G1 phase as well as mitochondrial fragmentation. This study identifies mitochondria-associated abnormalities in Bloom syndrome patient-derived and BLM-knockout cells and we discuss how these abnormalities may contribute to Bloom syndrome.

## Introduction

Bloom syndrome (BS; OMIM #210900; ORPHA #125) is a rare autosomal recessive disorder that is associated with a broad phenotypic spectrum including growth retardation, photosensitive skin, compromised immune system, insulin resistance, and high cancer predisposition, with some of these symptoms reminiscent of aging-associated phenotypes^1^. Average lifespan is 25 years with the most common cause of death being cancer^1^. Bloom syndrome is caused by loss of function mutations in the *BLM* gene (OMIM #604610), which encodes a 3′ to 5′ DNA helicase belonging to the evolutionarily conserved RecQ family^2^. BLM’s best characterized function is in the repair of DNA double-strand-breaks by homologous recombination where it is involved in DNA end resection, D-loop and Rad51 filament formation, branch migration and Holliday junction dissolution^3^. BLM also readily unwinds DNA secondary structures and its ability to unwind G-quadruplexes is one of the most efficient among human DNA helicases^4,5^.

Cells from patients with Bloom syndrome exhibit a variety of cytogenetic abnormalities, such as increased levels of chromatid breaks, mitotic recombination, sister chromatid exchanges (SCE), and quadriradial chromosomes^3,6^. This genome instability phenotype demonstrates multiple roles of the BLM protein in DNA recombination and repair. Although functional abnormalities observed in Bloom syndrome patients can be explained by genome instability or defects in the DNA damage response, previous reports also suggest that some characteristics of Bloom syndrome could result from oxidative stress. These include elevated spontaneous mutation rates, delayed DNA fork progression rate, impaired DNA repair enzyme activities, immunological disorders, insulin resistance and cancer predisposition^7–12^.

Bloom syndrome cells exhibit cellular redox alterations and exist in a pro-oxidant state in vivo^13^. Cells lacking BLM demonstrate enhanced levels of active oxygen radicals and elevated superoxide dismutase activity^14^. Additionally, studies conducted on Bloom syndrome patient clinical samples also revealed redox alterations as indicated by high concentrations of plasma uric acid and high levels of 8-oxo-dG in leukocytes^15^. Treatment of Bloom syndrome cells with lipid peroxidation inhibitor α-tocopherol was found to reduce the rates of sister chromatid exchanges^14^. Taken together, these findings suggest that BLM-deficient cells are in a perpetual state of oxidative stress and that the BLM helicase is important for maintaining cellular redox homeostasis. These studies are based on redox abnormalities observed in cultured cells from patients with Bloom syndrome or clinical studies based on patient samples. However, to date, the cause of this oxidative stress remains unclear. It is also not understood whether the oxidative stress phenotype observed in Bloom syndrome is due to overproduction of ROS or impairment of antioxidant defenses. Given that the basal levels of oxidative stress biomarkers are upregulated in BLM-deficient cells in the absence of any external ROS sources, the molecular events triggering oxidative stress phenotype are likely to be endogenous.

Most endogenous ROS are produced during metabolic reactions involving mitochondria^16^. Mitochondria are the ‘power plants’ of the cells where the bulk of the cellular ATP production takes place through oxidation of metabolic fuels by the electron transport chain and oxidative phosphorylation^17^. These processes produce significant amounts of ROS. Since mitochondria are the major cellular sites for generation of ROS as well as the main targets for their action, mitochondrial dysfunction has become a characteristic hallmark for many age-related pathologies like neurodegenerative diseases, cardiovascular diseases, metabolic syndrome and cancer^18^. Oxidative stress by mitochondrial ROS can contribute to genome instability including chromosome breaks, chromosomal translocations, end-to-end fusions and loss of cell viability and proliferative capacity in fibroblasts^19^. Similarly, mitochondrial dysfunction and increased ROS generation have been associated with telomere loss and chromosome damage in single cell zygotes^20^. The ROS generated from mitochondria can participate in mitochondrial stress signaling and also introduce mitochondrial/nuclear DNA mutations that can contribute to the initiation and amplification of the tumor cell phenotype^21^. Interestingly, cancer prone genetic disorders like Ataxia-Telangiectasia, Fanconi Anemia, and Werner syndrome show both oxidative stress and mitochondrial dysfunction as hallmarks of disease pathology^12,22–27^.

Here, we have investigated levels of endogenous oxidative stress in a Bloom-syndrome-patient-derived cell line and in cells with a CRISPR/Cas9-based *BLM* gene disruption, finding effects on DNA damage levels, DNA replication speed, mitochondrial mass, mitochondrial transcription factor A (TFAM) expression, and mitochondrial network morphology. Moreover, BLM-deficient cells fail to degrade cyclin B1 in early mitosis and G1, which was associated with persistent activation of mitochondrial fission protein Drp1 into G1 phase, providing a possible mechanism for mitochondrial fragmentation in BLM-deficient cells. Our observation that BLM-deficient cells have higher levels of ATP further prompted us to investigate the bioenergetic profile of BLM-deficient cells.

## Results

### Increased oxidative stress, DNA damage, and ROS-induced retardation of DNA replication speed in BLM*-*deficient fibroblasts

**Figure 1:**
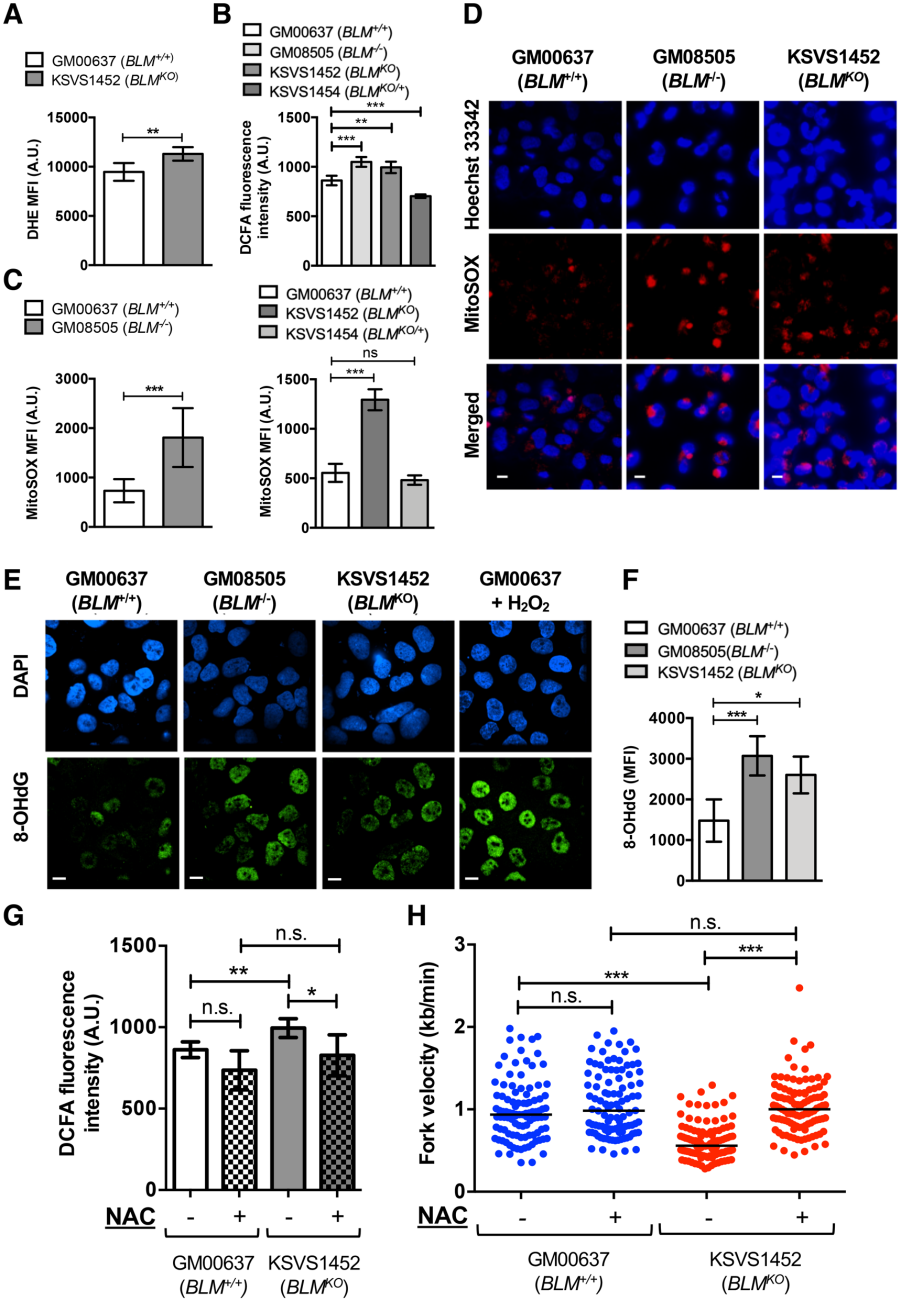
BLM-deficient cells exhibit high levels of ROS, oxidative DNA damage, and ROS-dependent reduction of DNA replication speed. (**A**) Flow cytometry measurement of cellular superoxide levels in GM00637 (*BLM*^+*/*+^) and KSVS1452 (*BLM*^*KO*^) cells stained with the super-oxide specific probe dihydroethidium (DHE). Analysis was performed in triplicate and mean ± SD is shown. (**B**) Fluorescence measurements of 2′,7′-dichlorofluorescin diacetate (DCFA)-stained BLM-proficient GM00637 (*BLM*^+*/*+^) cells, Bloom-syndrome-patient-derived GM08505 (*BLM*^*−/−*^) cells, *BLM*-knockout cells KSVS1452 (*BLM*^*KO*^) and *BLM*-complemented cells KSVS1454, indicating cellular ROS. Analysis was performed in triplicate and mean ± SD is shown. (**C**) Flow cytometry measurements of MitoSOX-stained (mitochondrial ROS) BLM-proficient GM00637 (*BLM*^+*/*+^) cells, Bloom-syndrome-patient-derived GM08505 (*BLM*^*−/−*^) cells, *BLM*-knockout cell line KSVS1452 (*BLM*^*KO*^) and *BLM*-complemented cell line KSVS1454. Analysis was performed in triplicate and mean ± SD is shown. (**D**) Representative fluorescence microscopy images of live GM00637 (*BLM*^+*/*+^), GM08505 (*BLM*^*−/−*^) and KSVS1452 (*BLM*^*KO*^) cells stained with MitoSOX (red foci), indicating mitochondrial ROS, and with Hoechst 33342 (blue). Scale bars 10 µm. (**E**) Confocal microscopy images of fixed GM00637 (*BLM*^+*/*+^), GM08505 (*BLM*^*−/−*^) and KSVS1452 (*BLM*^*KO*^) cells immunostained for oxidative damage to DNA (antibody 8-oxo-dG, 15A3, SCBT). GM00637 (*BLM*^+*/*+^) cells exposed to 100 µM H_2_O_2_ for 2 h served as a positive control for oxidative nucleotide damage. Scale bars 10 µm. (**F**) Median fluorescence intensity was determined by flow cytometry in fixed GM00637 (*BLM*^+*/*+^), GM08505 (*BLM*^*−/−*^) and KSVS1452 (*BLM*^*KO*^) cells immunostained with antibody 8-oxo-dG 15A3 (SCBT). Analysis was performed in triplicate and mean ± SD is shown. (**G**) Fluorescence measurements of 2′,7′-dichlorofluorescin diacetate (DCFA)-stained BLM-proficient GM00637 (*BLM*^+*/*+^) cells and *BLM*-knockout cells KSVS1452 (*BLM*^*KO*^) either treated with 5 mM antioxidant *N*-acetyl-cysteine (NAC) for 24 h, or untreated. Analysis was performed in triplicate and mean ± SD is shown. (**H**) Measurement of DNA replication speed in GM00637 (*BLM*^+*/*+^) cells and *BLM*-knockout cells KSVS1452 (*BLM*^*KO*^) either treated with 5 mM antioxidant N-acetyl-cysteine (NAC) for 24 h, or untreated, prior to DNA fiber analysis. Significance of differences was determined by a Student’s t-test and is reported as *p ≤ 0.05, **p ≤ 0.01, ***p ≤ 0.001; ns, not significant. Flow cytometry data were analyzed with FlowJo v. 10.7 software (BD Life Sciences, https://www.flowjo.com/solutions/flowjo/downloads).

To evaluate oxidative stress in BLM-deficient cells we first assessed levels of reactive oxygen species (ROS) production. In addition to analyzing a Bloom-syndrome-patient-derived cell line (*BLM*^*−/−*^, GM08505) we constructed a BLM-knockout cell line by CRISPR/Cas9-mediated disruption of *BLM* exon 8 in the human skin fibroblast cell line GM00637. The two *BLM* alleles in the resulting cell line KSVS1452 (*BLM*^*KO*^) contain different frameshift mutations that lead to termination at codon 686 upstream of conserved helicase motif I (allele 1*: BLM* c.del2040_2046; g.del90,763,123_90,763,130; BLM p.Leu681LysTer686; allele 2: *BLM* c.delins2036_2041AG; g.delins90,763,119_90,763,124AG; BLM p.Leu678AlaTer686). These mutations are upstream of the most common Bloom-syndrome-causing mutation in the human population, *BLM*^*Ash*^, which causes a frameshift mutation in exon 10 that leads to a premature stop in codon 740^2^. KSVS1452 shows no detectable BLM protein and exhibits cellular defects characteristic of the Bloom-syndrome-patient-derived cell line GM08505, including increased levels of sister-chromatid exchanges, hypersensitivity to DNA-damaging agents, delayed repair of replication-dependent DNA double-strand breaks, and increased doubling time (Supplemental Fig. S1A–F), confirming successful inactivation of *BLM* in KSVS1452. In addition to using the BLM-proficient cell line GM00637, we generated a stable BLM-proficient cell line by reintroducing BLM cDNA into KSVS1452 to serve as a control (*BLM*^*KO/*+^, KSVS1454; Supplemental Fig. S1G). By flow cytometry, we observed an increase in cellular ROS levels in KSVS1452 (*BLM*^*KO*^) using dihydroethidium (DHE) staining, which detects superoxide and hydrogen peroxide (Fig. 1A). In addition, we assessed cellular ROS using 2′,7′-dichlorofluorescin diacetate (DCFA), which is activated by most reactive oxygen species in cells. Both, the patient-derived GM08505 cells and the BLM-knockout KSVS1452 cells showed significantly increased ROS levels compared to the BLM-proficient GM00637 cells whereas BLM-complemented KSVS1454 cells showed normal ROS levels (Fig. 1B). Flow cytometric analysis with MitoSOX-Red showed elevated levels of mitochondrial superoxide, a major intracellular source of ROS, in patient-derived *BLM*^−/−^ cells and in *BLM*^*KO*^ cells whereas reintroduction of wildtype BLM cDNA into KSVS1452 (KSVS1454, *BLM*^*KO*/+^) reversed mitochondrial superoxide ROS levels to normal (Fig. 1C). This was consistent with fluorescence microscopy using MitoSOX staining, which showed increased formation of fluorescent puncta in the BLM-deficient cell lines (Fig. 1D).

One of the well-characterized base lesions induced by ROS that is involved in DNA mutagenesis is oxidized guanine nucleoside (8-oxo-dG)^28^. Using immunofluorescence microscopy and flow cytometry, we found that GM08505 (*BLM*^−/−^) and KSVS1452 (*BLM*^*KO*^) cells had significantly higher levels of 8-oxo-dG, indicating that BLM-deficiency increases oxidative base damage (Fig. 1E,F).

BLM-deficient cells do not exhibit gross cell cycle defects (Supplemental Fig. S2A), but they grow more slowly (Supplemental Fig. S1F) and show a slight accumulation of G1- and S-phase cells (Supplemental Fig. S2B). As ROS was recently also shown to affect DNA replication fork velocity^9,29^ we assessed whether ROS contributes to the decreased DNA replication speed observed in BLM-deficient cells^8,30^. Indeed, treatment of KSVS1452 (*BLM*^*KO*^) cells with the antioxidant *N*-acetyl-cysteine (NAC) significantly reduced cellular ROS (Fig. 1G) and increased DNA replication speed to levels seen in BLM-proficient cells (Fig. 1H). In contrast, NAC had no effect on DNA replication speed in BLM-proficient cells (Fig. 1H). This suggests that elevated ROS contributes to the slowing of DNA replication in BLM-deficient cells.

### Increased mitochondrial mass and TFAM upregulation in BLM-deficient cells

**Figure 2:**
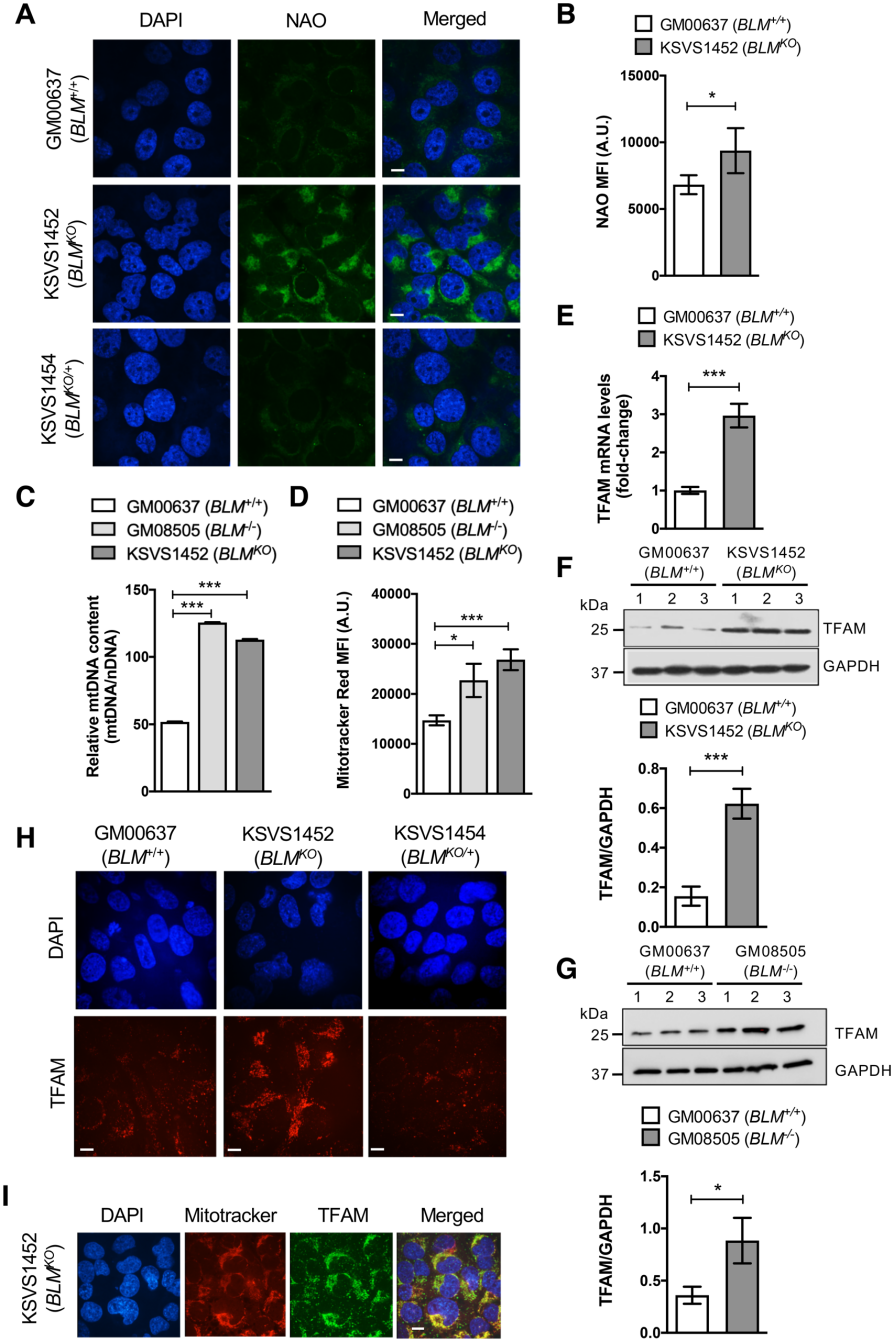
TFAM and mitochondrial mass are upregulated in BLM-deficient cells. (**A**) Confocal microscopy images showing NAO stained BLM-proficient GM00637 (*BLM*^*+/+*^) and KSVS1454 (*BLM*^*KO/*+^) cells and BLM-deficient KSVS1452 (*BLM*^*KO*^) cells. KSVS1454 (*BLM*^*KO/*+^) was generated from KSVS1452 (*BLM*^*KO*^) by stable transfection with BLM cDNA (Supplemental Fig. S1G). Scale bars 10 µm. (**B**) Flow cytometry analysis of mitochondrial staining of GM00637 (*BLM*^+/+^) and BLM-deficient KSVS1452 (*BLM*^*KO*^) cells with NAO. Analysis was performed in triplicate and mean ± SD is shown. (**C**) Relative mitochondrial DNA content measured by qPCR using primers against mitochondrial gene *COX1* and nuclear gene *18S*. Analysis was performed in triplicate and mean ± SD is shown. (**D**) Flow cytometry analysis of mitochondrial staining of GM00637 (*BLM*^+/+^) and BLM-deficient KSVS1452 (*BLM*^*KO*^) and GM08505 (*BLM*^−/−^) cells by membrane-potential-dependent Mitotracker Red CMXRos. Analysis was performed in triplicate and mean ± SD is shown. (**E**) TFAM mRNA levels in BLM-proficient (GM00637) cells and BLM-deficient (KSVS1452) cells were determined by qRT-PCR. (**F**–**G**) Western Blot analysis for TFAM expression levels in whole cell extracts from GM00637 (*BLM*^+/+^), the isogenic BLM-deficient cell line KSVS1452 (*BLM*^*KO*^) and in patient-derived GM08505 (*BLM*^−/−^) cell line. Whole cell extracts were prepared from three independent cultures and TFAM levels quantified from Western blots using Image J. GAPDH was used as a loading control. (**H**) Confocal microscopy images of fixed GM00637 (*BLM*^+/+^), KSVS1452 (*BLM*^*KO*^) and KSVS1454 (*BLM*^*KO/*+^) cells immunostained for endogenous TFAM. See also Fig. S3A for TFAM staining in GM08505 (*BLM*^−/−^) cells. Scale bars 10 µm. (**I**) Confocal microscopy images of KSVS1452 cells immunostained for TFAM and co-stained with Mitotracker and DAPI. Scale bars 10 µm. Significance of differences was determined by a Student’s t test and is reported as *p ≤ 0.05, **p ≤ 0.01, ***p ≤ 0.001. Flow cytometry data were analyzed with FlowJo v. 10.7 software (BD Life Sciences, https://www.flowjo.com/solutions/flowjo/downloads).

Quantitative PCR showed that mtDNA content is significantly increased in BLM-deficient cells (Fig. 2C). NAO, a fluorescent dye that localizes to cardiolipin in the inner mitochondrial membrane in a membrane-potential-independent manner^31^, also showed increased staining of BLM-deficient cells by fluorescence microscopy (Fig. 2A) and flow cytometry (Fig. 2B). Both, GM08505 (*BLM*^−/−^) and KSVS1452 (*BLM *^*KO*^) cells, also showed increased staining with MitoTracker CMXRos, which measures mitochondrial mass in a membrane-potential-dependent manner^31^ (Fig. 2D). Together, these findings suggest increased mitochondrial mass with intact membrane potential in BLM-deficient cells.

Mitochondrial transcription factor A (TFAM) binds to mtDNA, packaging it into nucleoids, and acts as a major factor in regulating mitochondrial transcription, replication and biogenesis^32,33^. TFAM was upregulated fourfold in KSVS1452 (*BLM*^*KO*^) cells (Fig. 2F) and 2.5-fold in Bloom- syndrome-patient-derived GM08505 (*BLM*^−/−^) cells (Fig. 2G), consistent with increased mitochondrial mass in these cells. Messenger RNA levels indicate transcriptional upregulation of TFAM in the absence of BLM (Fig. 2E). Immunofluorescence microscopy further demonstrated increased TFAM staining in BLM*-*deficient fibroblasts (Fig. 2H, Supplemental Fig. S3A) that was reduced to wildtype levels upon reintroduction of BLM cDNA (KSVS1454, Fig. 2H), verifying BLM-dependent changes in TFAM levels. The increased amount of TFAM found in BLM-deficient cells localized normally to mitochondria (Fig. 2I).

Levels of Nrf1, a key activator of TFAM expression^34^, were slightly upregulated in BLM-deficient KSVS1452 cells whereas Nrf2, which also contributes to TFAM induction^34^, did not change significantly (Supplemental Fig. S3B,C), suggesting that oxidative stress regulators make some contribution to TFAM upregulation in BLM-deficient cells. Besides Nrf1/Nrf2, Myc also binds to the *TFAM* gene and upregulates its expression^35^. Interestingly, c-myc levels are increased in BLM-deficient cell lines and BLM negatively regulates c-myc by promoting c-myc degradation through the E3 ubiquitin ligase FBW7^36–39^. Thus, increased myc levels may provide an additional mechanism for TFAM upregulation in BLM-deficient cells.

### Increased mitochondrial mass is not due to defective autophagy

**Figure 3:**
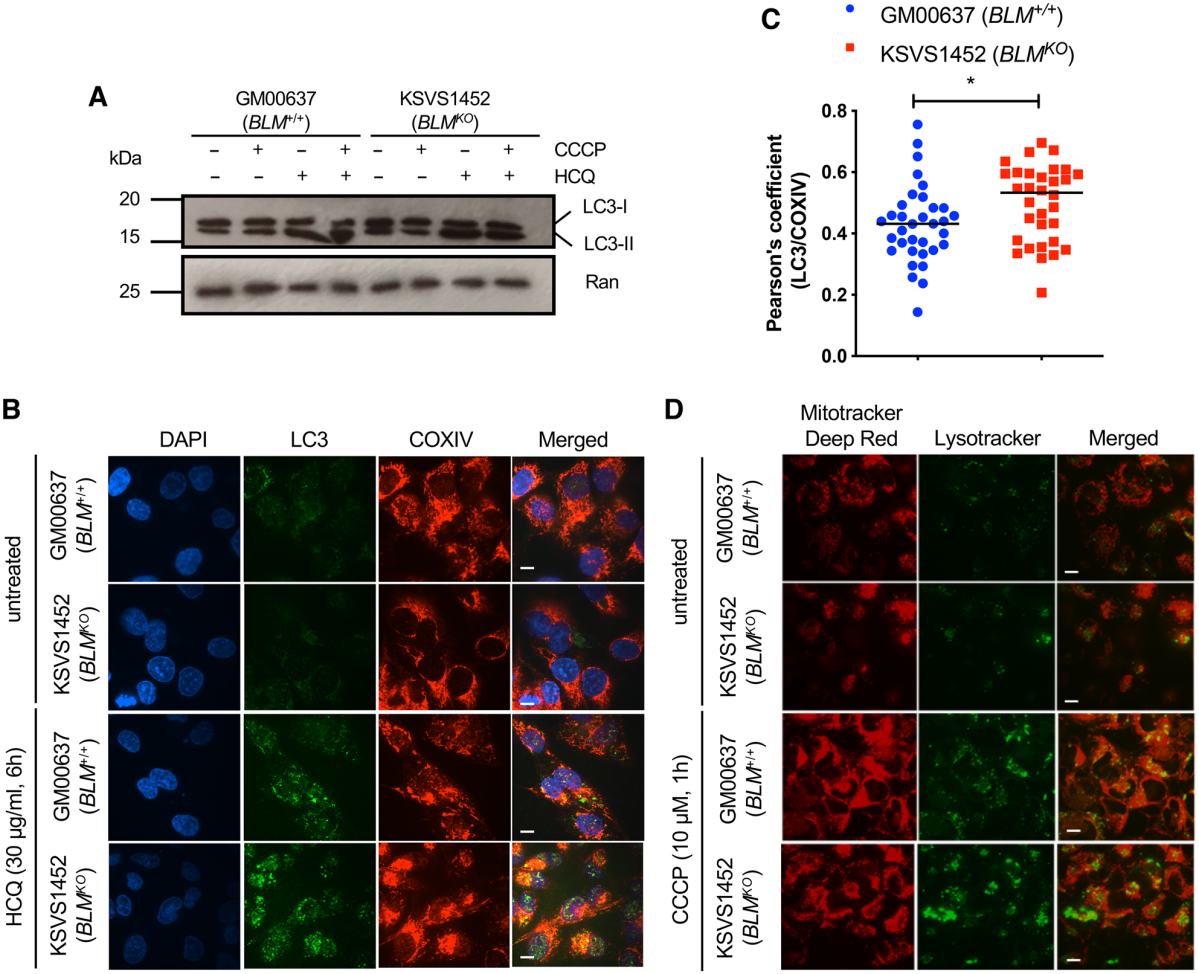
BLM-deficient cells are proficient in autophagy. (**A**) Western blot analysis of GM00637 (*BLM*^+/+^) and KSVS1452 (*BLM*^*KO*^) cells for levels of cytosolic LC3-I and autophagosomal marker LC3-II. Both, cytosolic LC3-I and membrane-bound LC3-II, were detected with LC3 antibody G-9 (SCBT). Cells were treated with 10 µM CCCP for 6 h to induce autophagy and/or with 30 µg/ml HQC for 6 h to interrupt autophagic flux. (**B**) Confocal microscopy images of fixed GM00637 (*BLM*^+/+^) and KSVS1452 (*BLM*^*KO*^) cells immunostained for LC3 (MAP LC3β-G-9 (SCBT) and COXIV (Cell Signaling), and stained with DAPI. Cells were treated with 30 µg/ml HCQ for 6 h to interrupt autophagic flux. Scale bars 10 µm. (**C**) Quantification of colocalization of antibody staining for LC3 and COXIV in HCQ-treated cells imaged as in panel B. 30 cells from BLM-proficient GM00637 cells and BLM-deficient KSVS1452 cells were analyzed. Significance of differences was determined by a Student’s t test and is reported as *p ≤ 0.05. (**D**) Confocal microscopy images of BLM-proficient GM00637 cells and isogenic BLM-knockout cells KSVS1452 stained with MitoTracker Deep Red and Lysotracker in the presence and absence of CCCP. Scale bars 10 µm.

An increase in mitochondrial mass could reflect changes in mitophagy^40^. To determine whether autophagic activity is impaired in cells lacking BLM, we measured changes in the levels of cytosolic LC3-I and autophagosomal marker LC3-II in the presence of the mitochondrial oxidative phosphorylation uncoupler CCCP and treatment with hydroxychloroquine (HCQ), which blocks autophagosome-lysosome fusion^41^. Treatment with CCCP induces autophagy and conversion of LC3-I to membrane-bound LC3-II whose accumulation can be visualized by blocking autophagic flux with HCQ. We observed that upon HCQ treatment LC3-II readily accumulated in both BLM-proficient GM00637 cells and BLM-deficient KSVS1452 cells, indicating normal autophagic flux (Fig. 3A). We further analyzed autophagic flux by immunofluorescence microscopy using double staining with antibodies against LC3 (autophagosome) and COXIV (mitochondria) (Fig. 3B). LC3 is recruited to the membranes of autophagosomes forming punctate structures. The number of LC3 puncta increased in BLM-proficient and BLM-deficient cells after HCQ exposure, indicating functional autophagy in both cell lines. LC3 foci colocalized with mitochondria in both cell lines; the slight increase in colocalization seen in the BLM-deficient KSVS1452 cells (Fig. 3C) could be due to the increased mitochondrial mass in these cells or indicate increased mitophagy. Finally, after treatment of BLM-proficient and BLM-deficient cell lines with the mitochondrial oxidative phosphorylation uncoupler CCCP, mitochondria localized to lysosomes (Fig. 3D). Overall, these findings indicate that mitophagy is not impaired in cells lacking BLM.

### BLM deficiency is associated with mitochondrial fragmentation

**Figure 4:**
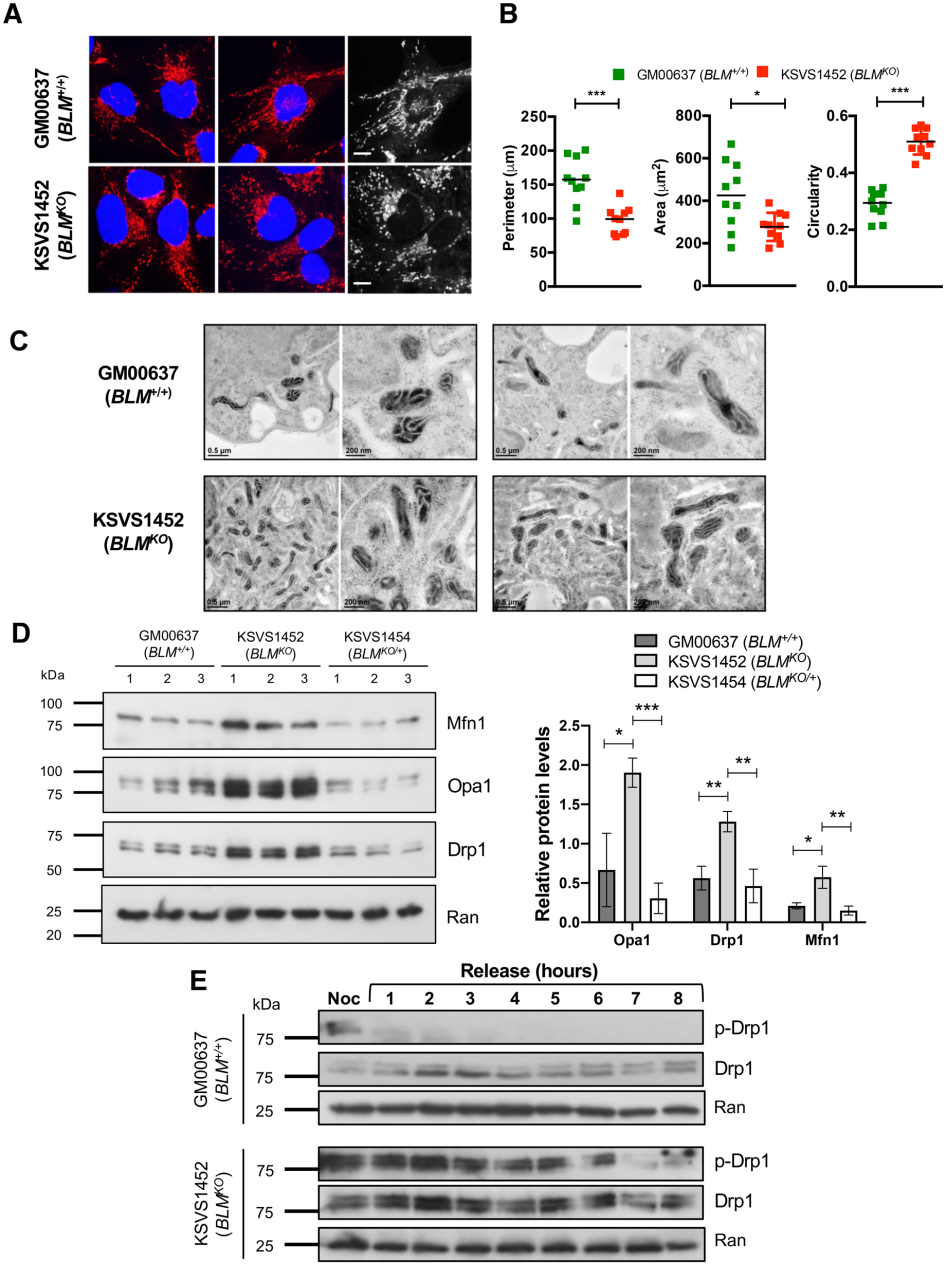
BLM deficiency is associated with mitochondrial fragmentation and persistent activation of mitochondrial fission protein Drp1. (**A**) Confocal microscopy images showing mitochondrial morphology in fixed GM00637 (*BLM*^+/+^) and BLM-deficient KSVS1452 (*BLM*^KO^) cells stained with Mitotracker (red) and DAPI (blue). Scale bars 10 µm. (**B**) Measurements of mitochondrial network morphology parameters using the Mito-Morphology Macro^91^ in ImageJ (version 1.53a; freely available at http://imageJ.nih.gov/ij). (**C**) Mitochondria of cells prepared by high-pressure freezing/freeze substitution fixation (HPF/FS) were imaged by transmission electron microscopy. Sections of two representative cells of each cell line are shown at higher (scale bar 200 nm) and lower (scale bar 0.5 µm) magnification. Images were obtained with Digital Micrograph software v. 1.93.1362 (Gatan Microscopy Suite (GMS) software, https://www.gatan.com/installation-instructions#Step1) (**D**) Western blot analysis and quantification of expression levels from whole cell extracts of fission marker Drp1 ([6Z-82], SCBT) and fusion markers Mfn1 ([D-10], SCBT) and Opa1 ([612606], BD Biosciences) in BLM-proficient cell lines GM00637 (*BLM*^+/+^) and KSVS1454 (*BLM*^*KO/*+^) and in BLM-deficient KSVS1452 (*BLM*^KO^) cells. The three cell lines are isogenic; KSVS1454 (*BLM*^*KO/*+^) was generated from KSVS1452 (*BLM*^KO^) by stable transfection with BLM cDNA (Fig. S1G). Whole cell extracts were prepared from three independent cultures and quantification was performed using ImageJ (version 1.53a; freely available at http://imageJ.nih.gov/ij). Ran ([610340], BD Biosciences) was used as a loading control. (**E**) Western blot analysis of Drp1 phosphorylation status in the BLM-proficient GM00637 (*BLM*^+*/*+^) and BLM-deficient KSVS1452 (*BLM*^*KO*^) cell lines following G2/M arrest and release. Cells were blocked at G2/M with nocodazole, released for 8 h and expression levels and phosphorylation status of Drp1 analyzed by Western blot using Drp1 antibody 6Z-82 (SCBT) and phospho-Drp1-Ser616 antibody D9A1 (Cell Signaling). Ran ([610340], BD Biosciences) was used as a loading control. Significance of differences was determined by a Student’s t test and is reported as *p ≤ 0.05, **p ≤ 0.01, ***p ≤ 0.001.

Since BLM deficiency is associated with increased mitochondrial mass we evaluated the effect of BLM loss on the regulation of mitochondrial network dynamics. Throughout most of the cell cycle, mitochondria form a filamentous network, which is fragmented at the transition into mitosis to allow for the distribution of mitochondria into the daughter cells, linking mitochondrial fission to cell division^42^. On performing fluorescence microscopy with Mitotracker Red CMXRos, we observed that the mitochondrial network in BLM-deficient cells was fragmented (Fig. 4A). Specifically, mitochondria in BLM-deficient cells were significantly smaller, more circular, and less branched than mitochondria in BLM-proficient GM00637 cells (Fig. 4B). Electron microscopy showed no swelling or other gross abnormalities in the inner structure of mitochondria of BLM-deficient cells (Fig. 4C).

Mitochondrial fission is mediated by the dynamin-like GTPase Drp1 whose phosphorylation at serine 616 leads to self-assembly into spirals that constrict the mitochondrial tubules, leading to fission^43^. BLM-deficient KSVS1452 cells exhibited a significant increase in Drp1 levels, which returned to normal upon reintroduction of BLM cDNA (KSVS1454) (Fig. 4D). However, fusion proteins Opa1 and Mfn1 were also upregulated, suggesting that mitochondrial fission and fusion protein levels are generally increased in BLM-deficient cells due to increased mitochondrial mass (Fig. 4D). Drp1 fission activity is regulated by phosphorylation, including at the transition to mitosis where it mediates mitochondrial fission prior to cell division^44^. To assess possible differences in Drp1 activity in BLM-deficient cells, we blocked cells at G2/M with a single thymidine arrest followed by nocodazole block and assessed Drp1-S616 phosphorylation after release into mitosis and the subsequent G1 phase over an 8-h time course (Fig. 4E). As expected, Drp1 was rapidly dephosphorylated after entry into mitosis in wildtype cells; however, in *BLM*^*KO*^ cells Drp1 phosphorylation persisted throughout mitosis and into G1 phase. These findings suggest that persistent Drp1 activation could be responsible for the mis-timed mitochondrial fragmentation in KSVS1452 (*BLM*^*KO*^) cells.

### Unscheduled Cyclin B1 expression in BLM-deficient cells in late mitosis and G1

**Figure 5:**
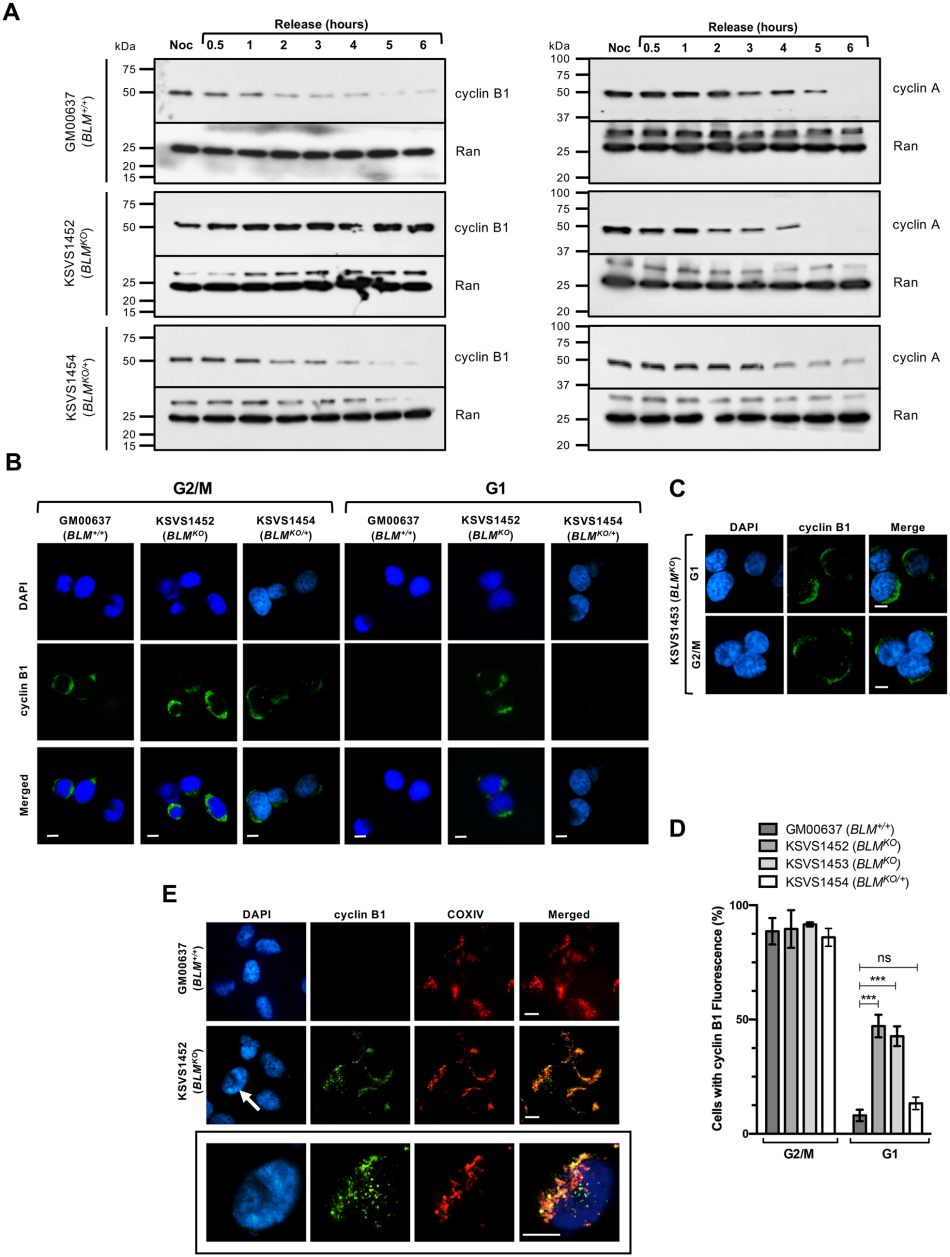
BLM-deficient cells exhibit unscheduled cyclin B1 expression during G1 phase that localizes to mitochondria. (**A**) Western Blot analysis of expression levels of cyclin B1 ([GNS1], SCBT) and cyclin A ([BF683], SCBT) in whole cell extracts of BLM-proficient cell lines GM00637 (*BLM*^+/+^) and KSVS1454 (*BLM*^*KO/*+^) and BLM-deficient cell line KSVS1452 (*BLM*^*KO*^) following G2/M block and release. Ran ([610340], BD-Biosciences) was used as a loading control. Cells were arrested with nocodazole, released into drug-free media and processed for Western blotting at the indicated time points. (**B**) Immunofluorescence microscopy of sorted G1 and G2/M phase cells of GM00637 (*BLM*^+*/*+^), KSVS1452 (*BLM*^*KO*^) and KSVS1454 (*BLM*^*KO/*+^). KSVS1454 is the *BLM*-complemented KSVS1452 cell line. Sorted cells were immunostained for cyclin B1 ([GNS1], SCBT) and counterstained with DAPI. Scale bars 10 µm. (**C**) Immunofluorescence microscopy for cyclin B1 staining was repeated for G1 and G2/M sorted cells of KSVS1453, an independent second clone of the biallelic BLM-knockout cell line. (**D**) Percentage of sorted G1 and G2/M cells of BLM-proficient cell lines [GM00637 (*BLM*^+*/*+^), KSVS1454 (*BLM*^*KO/*+^)] and BLM-deficient cell lines [KSVS1452 (*BLM*^*KO*^), KSVS1453 (*BLM*^*KO*^)] cells exhibiting cyclin B1 fluorescence. A minimum of 300 cells from three independent cultures of each cell line was analyzed by confocal microscopy. Mean ± SD is reported. (**E**) Confocal microscopy images of fixed sorted G1 phase cells of GM00637 (*BLM*^+*/*+^) and KSVS1452 (*BLM*^*KO*^) immunostained for cyclin B1 ([GNS1], SCBT) and COXIV ([3E11], Cell Signaling) and counterstained with DAPI. Bottom panel shows an enlargement of the nucleus indicated by a white arrow. Scale bars 10 µm. Significance of differences in (**D**) was determined by a Student’s t-test and is reported as ***p ≤ 0.001; ns, not significant.

One of the key phosphorylation events that activates Drp1 is mediated by cyclin B1/CDK1 during transition into mitosis^45^. To determine if persistent Drp1 phosphorylation in BLM*-*deficient cells is associated with changes in levels of cyclin B1/CDK1, we blocked GM00637 (*BLM*^+/+^) and KSVS1452 (*BLM*^*KO*^) cells at G2/M with nocodazole, collected samples for 6 h after release and assessed degradation of cyclin B1 (Fig. 5A). Normally, cyclin B1 is highly expressed in late S and G2 phase to form the cyclin B1/CDK1 kinase complex where its function during the G2/M transition, along with Drp1 phosphorylation, is to target and phosphorylate all five-major multi-subunit respiratory complexes of the electron transport chain. This phosphorylation corresponds with a dramatic increase in mitochondrial respiration and ATP production, which allows cells to overcome the large energy expenditure of mitosis^46^. Cyclin B1 is then degraded as cells progress through mitosis following the inactivation of the spindle assembly checkpoint and entry into anaphase^47^. Here, we observed that cyclin B1 was degraded with the expected kinetics in the GM00637 (*BLM*^+/+^) cell line, whereas even after 6 h following the release from nocodazole block, cyclin B1 levels remained unchanged in BLM-deficient KSVS1452 (*BLM*^*KO*^) cells (Fig. 5A). In contrast, the degradation pattern of cyclin A, which is also degraded during mitosis^47^, was similar in both cell lines, indicating proper re-entry into the cell cycle after release from nocodazole block (Fig. 5A). To verify that sustained cyclin B1 levels were associated with the loss of BLM, we assessed cyclin B1 degradation in the BLM-complemented KSVS1454 (*BLM*^*KO/*+^) cell line. Indeed, cyclin B1 degradation in KSVS1454 cells returned to the normal pattern seen in GM00637 (*BLM*^+/+^) cells (Fig. 5A). We confirmed normal mitotic re-entry and progression into G1 after release from nocodazole by flow cytometry in all three cell lines (Supplemental Fig. S4A).

To eliminate the possibility that the prolonged presence of cyclin B1 in the BLM-deficient cells stemmed from inefficient release into G1 phase or that it was related to the synchronization procedure we sorted asynchronous GM00637 (*BLM*^+/+^), KSVS1452 (*BLM*^*KO*^) and KSVS1454 (*BLM*^*KO/*+^) cultures into G1 and G2/M populations based on DNA content by flow cytometry (Supplemental Fig. S4B) and analyzed for cyclin B1 staining by immunofluorescence microscopy (Fig. 5B). As expected, all three cell lines exhibited cyclin B1 signals in their G2/M populations (Fig. 5B), reaching 89–91% of all cells (Fig. 5D). However, cyclin B1 expression was observed in 47% of the G1 population of the BLM-deficient KSVS1452 (*BLM*^*KO*^) cells in comparison to only 8% and 13% of the BLM-proficient GM00637 (*BLM*^+*/*+^) and KSVS1454 (*BLM*^*KO/*+^) cells, respectively (Fig. 5D). We confirmed unscheduled cyclin B1 expression in G1 phase in KSVS1453 (*BLM*^*KO*^) cells, a second independent clone of CRISPR/Cas9-mediated BLM disruption in GM00637 (Fig. 5C,D). Thus, cyclin B1 escapes mitotic degradation and is abnormally expressed during G1 phase in BLM-deficient cells. This abnormal cyclin B1 expression pattern in mitosis and in G1 is fully reversible by reintroducing BLM. Cyclin B1 localizes to mitochondria during the G2/M transition to upregulate ATP production for mitosis^46^. The vast majority of cyclin B1 untimely expressed during G1 in KSVS1452 (*BLM*^*KO*^) cells also localized to mitochondria, as indicated by overlap with the mitochondria-specific protein COXIV, whereas some cyclin B1 formed non-mitochondrial foci (Fig. 5E).

### Bioenergetic profile of BLM-deficient cells

**Figure 6:**
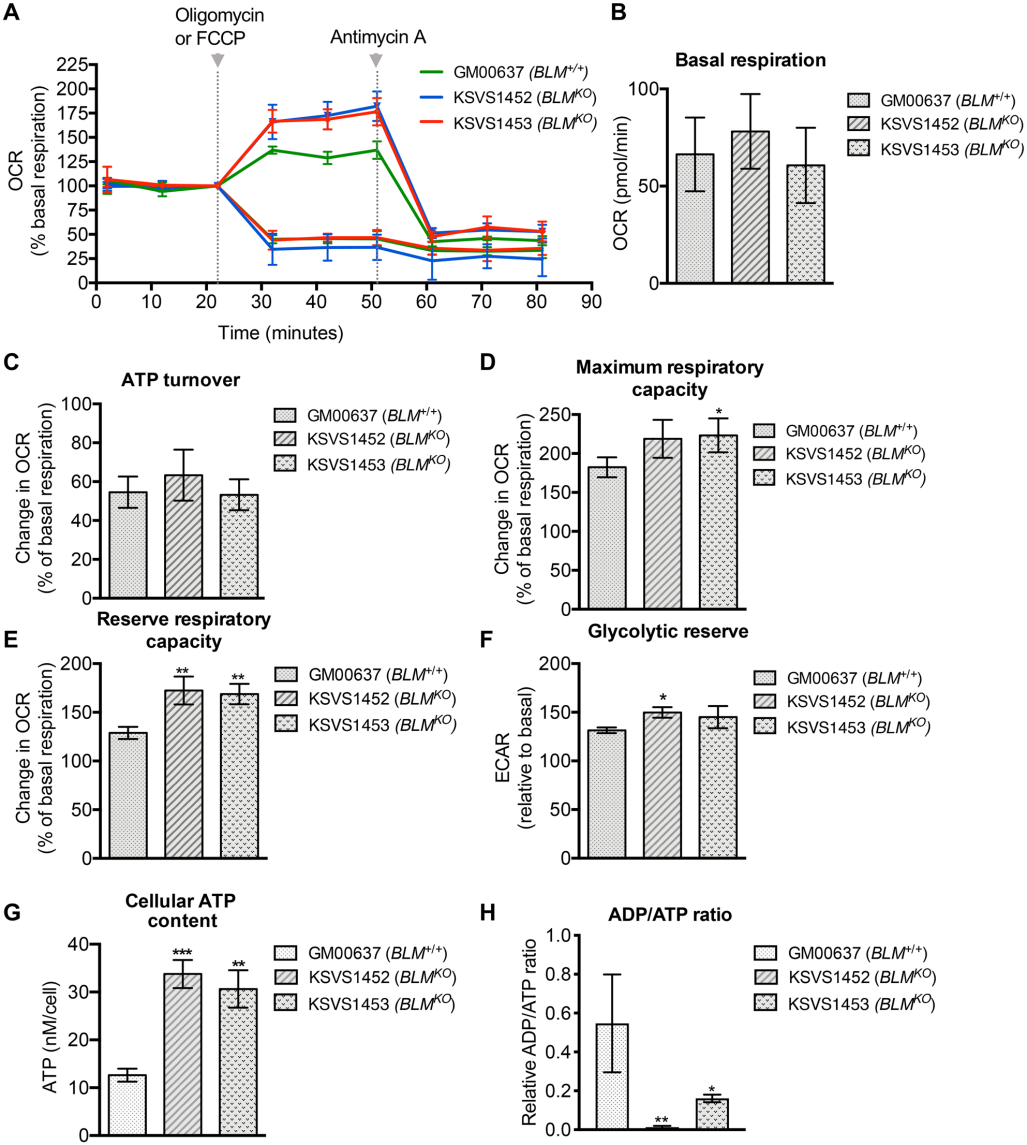
Bioenergetic profile of BLM-deficient cells. *(A*) Oxygen consumption rates (OCR) of BLM-proficient cell line GM00637 and BLM-deficient cell lines KSVS1452 and KSVS1453 were measured on a XF-96 Extracellular Flux Analyzer (Seahorse Bioscience) after addition of oligomycin or FCCP at 22 min and after addition of antimycin A after 51 min. From measurements in (**A**) basal respiration, (**B**), ATP turnover rate (**C**), maximum respiratory rate (**D**) reserve respiratory capacity (**E**) and glycolytic reserve (**F**) were calculated. All measurements were normalized to basal rate (data point at 22 min). (**G**) Total cellular ATP content was measured in whole cell extracts of BLM-proficient cell (GM00637) and BLM-deficient cells (KSVS1452, KSVS1543) using a luciferase-based assay. (**H**) ADP/ATP ratio was measured in whole cell extracts of BLM-proficient cells (GM00637) and BLM-deficient cells (KSVS1452, KSVS1453) using a bioluminescent method based on the conversion of ATP by luciferase.

To evaluate the function of the mitochondria of BLM-deficient cells we performed respiratory assays using well-defined mitochondrial inhibitors (Fig. 6A). We found no significant difference in basal oxygen consumption rate (OCR) between the BLM-proficient GM00637 cells and the two isogenic, independent BLM-knockout cell lines KSVS1452 and KSVS1453 (Fig. 6B). This allowed us to normalize ATP turnover, reserve respiratory capacity and maximal respiratory capacity to basal respiration to decrease growth-rate-related variability. ATP turnover as assessed by adding oligomycin, an inhibitor of complex V (ATP synthase) of the electron transport chain, demonstrated equimolecular oxygen use for production of ATP by oxidative phosphorylation in the BLM-proficient and BLM-deficient cells (Fig. 6C). Next, we added FCCP, an uncoupler that disrupts the mitochondrial membrane potential, to obtain the maximum oxygen consumption rate and the respiratory reserve capacity (Fig. 6D,E). Reserve respiratory capacity is the theoretical extra capacity the electron transport chain has to drive oxidative phosphorylation under situations of ATP need. Interestingly, the two BLM-deficient cell lines had a 30% higher reserve respiratory capacity than the isogenic BLM-proficient cell line (Fig. 6E). Taken together with identical ATP turnover rates in the BLM-proficient and BLM-deficient cell lines, this indicates that compared to the BLM-proficient cells the energy expenditure of BLM-deficient cells is lower than their rate of ATP synthesis. This is also reflected in the significantly higher (2.5-fold) cellular ATP content of the BLM-deficient cell lines compared to the isogenic BLM-proficient cell line (Fig. 6G) and an ADP/ATP ratio significantly shifted towards ATP in the BLM-deficient cell lines compared to the BLM-proficient cell line (Fig. 6H). Finally, we observed a trend towards a higher glycolytic reserve in BLM-deficient cells, which is a measure of the compensatory increase in glycolysis following an inhibition of the ATP synthase (Fig. 6F). Together, these findings indicate that BLM-deficient cells have a higher ability to produce ATP by oxidative phosphorylation than BLM-proficient cells under energy-requiring conditions and that they may be able to compensate for a loss of oxidative phosphorylation better than BLM-proficient cells. Overall, our analysis of mitochondrial function suggests that the BLM-deficient cell lines are not energetically impaired under the tested conditions and have a larger potential to produce ATP than the BLM-proficient cell line.

## Discussion

In this study, we have identified abnormalities in Bloom-syndrome-patient-derived cells and BLM-knockout cells that include increased ROS that causes DNA base damage and reduced DNA replication speed, increased mitochondrial mass and TFAM expression, and mitochondrial fragmentation. BLM-deficient cells also had higher ATP content and increased reserve respiratory capacity, but an otherwise unremarkable bioenergetic profile that was similar to the isogenic BLM-proficient parental cell line. Although the cause of mitochondrial network fragmentation in BLM-deficient cells is unknown, it is associated with untimely cyclin B1 expression into G1 phase and persistent activation of Drp1 at S616, which promotes mitochondrial fission at mitosis entry.

Upregulation of TFAM is a characteristic marker of many different cancer tissues and is associated with malignant progression and poor prognosis^48–52^, raising the possibility that it also contributes to cancer risk in Bloom syndrome. In addition to its role in cell proliferation and mitochondrial function, TFAM has been shown to regulate apoptosis. In certain types of cancer, TFAM binds to the promoters of apoptosis-related genes and regulates their expression^53^. TFAM depletion induces p21-dependent G1 arrest and p21-deficient cells exhibit elevated TFAM expression levels^53,54^. Thus, TFAM upregulation in BLM-deficient cells might also provide a mechanism to stimulate cell proliferation and prevent apoptosis to survive the defects caused by the absence of BLM activity.

In addition to increased TFAM expression and mitochondrial mass, we observed increased production of mitochondrial ROS in BLM-deficient cells. At low non-toxic concentrations, ROS play a role in signal transduction, but at higher levels they cause oxidative stress characterized by DNA base oxidation, lipid peroxidation and disruption of cellular redox status^38^. Unlike the human RecQ-helicase family member RecQ4, BLM does not localize to mitochondria^55^. Instead, increased ROS in BLM-deficient cells could be the result of the increased mitochondrial mass, but could also indicate changes in the arrangement of electron transport chain complexes in the mitochondrial membrane as a result of increased mitochondrial fragmentation. Indeed, increased ROS production has been shown to coincide with increased mitochondrial fragmentation^56–59^. Local cellular concentrations of ROS can lead to translocation of mitochondria to distinct cellular regions for signaling functions^60^. For example, the superoxide generating chemical MEN causes mitochondrial redistribution from the cell periphery to the perinuclear region^61^. Similarly, hypoxia causes perinuclear mitochondrial clustering^60^. Thus, high levels of ROS in BLM-deficient cells might contribute to the increased mitochondrial fragmentation and perinuclear clustering that we observed. Interestingly, such clustering of mitochondria around the nuclear periphery increases ROS levels in the nucleus^60^ and may contribute to the oxidative DNA damage we observed in BLM-deficient cells here as well as to the reduced DNA replication speed in Bloom syndrome patient cells previously reported^8,30^.

Remarkably, we found that normal DNA replication speed can be restored in Bloom syndrome cells by treating cells with an antioxidant, whereas antioxidant treatment had no effect on DNA replication speed in BLM-proficient cells. ROS could decrease DNA replication speed by generating DNA lesions that block DNA polymerases^62^, by oxidizing proteins involved in replication^63–66^, or by reducing the nucleotide pool^9^. Notably, replication fork velocity was recently shown to be coupled to ROS signaling through the fork accelerator TIMELESS-TIPIN^29^. As shown here for endogenous ROS-induced replication slowdown in BLM-deficient cells, NAC reverses slow replication speed induced by exposure to exogenous H_2_O_2_ whereas fork slowdown due to other causes, such as treatment with aphidicolin, is not affected by NAC^29^. Besides changing replication dynamics, increased nuclear ROS also induces transcriptional changes^67^, which could contribute to the risk of malignant transformation of Bloom syndrome cells.

The maintenance and networking of mitochondria is mediated by mitochondrial fusion and fission events and disruption of this balance is associated with developmental defects, neurodegeneration, metabolic diseases and aging^44,68–72^. In BLM-deficient cells, increased mitochondrial fragmentation was associated with persistent phosphorylation of the fission protein Drp1 whereas mitophagy appeared normal. The major phosphorylation event that activates Drp1 during the G2/M transition occurs at serine 616 by cyclin B1/CDK1 and links mitochondrial fission to cell division^42,45^. BLM-deficient cells failed to fully degrade cyclin B1 at anaphase and inappropriately accumulate cyclin B1 during G1, which is mostly localized to mitochondria. It is unclear how much of the constitutive mitochondrial fragmentation phenotype exhibited by BLM-deficient cells can be attributed to unscheduled cyclin B1 expression and oxidative-stress-induced fragmentation by Drp1^42,45^. However, any aberration in mitochondrial fission kinetics is likely to be problematic for proliferating cells. Mitochondrial fragmentation is observed in tumor cells and is associated with increased invasiveness and metastatic potential^73,74^. Drp1-induced mitochondrial fragmentation, such as seen here in BLM-deficient cells, also promotes tumor growth^75^. Mitochondrial dysfunction and mitochondrial fragmentation are also associated with insulin resistance, pancreatic β-cell dysfunction and development of Type 2 diabetes^70,76,77^. While we currently have no evidence that the mitochondrial abnormalities in BLM-deficient cells are associated with a loss of mitochondrial function, it is worth noting that ~ 17% of persons with Bloom syndrome develop Type 2 diabetes at a young age^78^.

An important function of the cyclin B1/CDK1 kinase complex during the G2/M transition is to target and phosphorylate all five major multi-subunit respiratory complexes of the electron transport chain to increase ATP production for mitosis^46,79^. It is plausible that the unscheduled mitochondrial cyclin B1 expression in G1 performs the same function and contributes to the increase in total ATP content in BLM-deficient cells. Since the G1/S and G2/M transition checkpoints are energy sensitive, it will be interesting to determine in future studies whether BLM-deficient cells require these increased levels of ATP for survival and if they compromise cell cycle processes and genomic stability*.*

Unscheduled cyclin B1 expression during G1 is observed in a growing number of cancer cell lines as well as in patient-derived primary cells from a broad range of tumor types^80,81^. The mechanisms underlying unscheduled appearance of cyclin B1 in G1 or its ability to escape normal degradation during the metaphase to anaphase transition have yet to be identified. However, cyclin B1 mRNA and translation appear to persist after mitosis, suggesting that rapid proteolysis is the more important mechanism for clearing cyclin B1 during G1 phase^82^. Similarly, in yeast, cyclin B1 degradation continues into G1^83^. It is unclear why this degradation process is impaired in the absence of BLM. One possibility is that the mitochondrial location of unscheduled cyclin B1 in G1 phase makes it less accessible to the G1 phase degradation system. This also raises the possibility that unscheduled cyclin B1 expression in late mitosis and in G1 phase is linked to the mitochondrial abnormalities in BLM-deficient cells. Indeed, not only is abnormal cyclin B1 expression associated with tumorigenesis, evidence for a link between the mitochondrial fragmentation phenotype and promotion of tumor growth is also increasing^75,84–86^.

Determining the bioenergetic profile of BLM-deficient cells revealed that their mitochondrial function was not impaired. On the contrary, their ATP levels were increased 2.5-fold and respiratory reserve capacity was increased by 30% compared to the isogenic BLM-proficient cell line. These differences suggest that BLM-deficient cells use less ATP than they produce, possibly due to their slower growth rate (Supplemental Fig. S1F), and have a higher capacity to respond to events of higher energy demand. Although we do not yet know the source of the increased respiratory reserve capacity in BLM-deficient cells, it is generally associated with increased cell survival under challenging conditions and may present a mechanism by which BLM-deficient cells cope with high levels of genome instability.

Even though it has been more than 60 years since Bloom syndrome was first described, the molecular mechanisms underlying some of its features have remained unclear^1^. As genome instability, cellular aging, degeneration and cancer predisposition have been linked to ROS, we propose that endogenous ROS overproduction, mitochondrial network abnormalities, especially as they relate to fission and fusion, and unscheduled cyclin B1 expression may contribute to the development of some symptoms of Bloom syndrome. Further studies will be needed to assess the potential pathogenicity of the mitochondrial abnormalities and cyclin B1 dysregulation and to determine if mitochondrially targeted antioxidants and free radical scavengers could serve as a prophylactic strategy to delay the onset of clinical symptoms in persons with Bloom syndrome. Inhibitors of mitochondrial fission are also being investigated as therapeutics for diseases with a mitochondrial fission phenotype and oxidative stress^87^. In addition to the well-understood role of BLM in homologous recombination and the DNA damage response, this study suggests that BLM may have additional roles in cell cycle processes and mitochondrial biogenesis through which it contributes to normal cell function.

## Materials and methods

### Cell culture and stable cell lines

SV40-transformed normal skin fibroblast cell line GM00637 (*BLM*^+/+^) and Bloom syndrome patient fibroblast cell line GM08505 (*BLM*^−/−^) were obtained from Coriell Cell Repository. The biallelic *BLM*-knockout cell line KSVS1452 (*BLM*^*KO*^) was derived from the GM00637 using a CRISPR/Cas9 mediated genome engineering protocol^88^. Guide RNA sequences targeting exon 8 of *BLM* gene were designed using a CRISPR guide design tool (http://tools.genome-engineering.org), cloned into pSpCas9 (BB)-2A-Puro and used for transfection of GM00637. *BLM*-knockout clones were screened by immunoblotting and biallelic gene disruptions verified by sequencing of both *BLM* alleles (KSVS1452 *BLM* allele 1*: BLM* c.del2040_2046; g.del90,763,123_90,763,130; BLM p.Leu681LysTer686; *BLM* allele 2: *BLM* c.delins2036_2041AG; g.delins90,763,119_90,763,124AG; BLM p.Leu678AlaTer686). The cellular phenotype of KSVS1452 was confirmed to resemble that of Bloom-syndrome-patient-derived cells as we have described before^89^ (Supplemental Fig. S1). KSVS1453 is a second, independent clone obtained by CRISPR/Cas9-mediated disruption of *BLM* exon 8. KSVS1452 was transfected with a plasmid expressing wildtype *BLM* cDNA (pcDNA3-BLM) and a stable clone selected to yield KSVS1454 (*BLM*^*KO/*+^). BLM expression levels in KSVS1454 corresponding to those of GM00637 cells were verified by Western blot (Supplemental Fig. S1G). Cells were grown in Modified Eagle’s Medium (MEM) supplemented with 10% Fetal Bovine Serum (FBS), 2 mM l-glutamine and 1% penicillin–streptomycin-glutamine (PSG) at 37 °C in the presence of 5% CO_2_.

### Immunoblotting

To prepare whole cell extracts from exponentially growing cultures, cells were lysed in RIPA lysis buffer [50 mM Tris, pH 8.0; 150 mM NaCl, 5 mM EDTA (pH 8.0), 1% NP-40, 0.5% sodium deoxycholate, 0.1% SDS and protease inhibitor cocktail (Pierce)]. Lysates were cleared by centrifugation at 12,000 rpm for 15 min and the supernatant was used for Western Blotting. Antibodies for immunoblotting were: TFAM [D5C8] (Cell Signaling), Drp1 [6Z-82] (SCBT), phospho-Drp1-Ser616 [D9A1] (Cell Signaling), cyclin B1 [GNS1] (SCBT), cyclin A [BF683] (SCBT), Ran [610340] (BD Biosciences), MAP LC3β [G-9] (SCBT), Mfn1 [D-10] (SCBT), GAPDH [GA1R] (Invitrogen), Opa1 [612606] (BD Biosciences), Nrf1 [147.1] (SCBT) and Nrf2 [A-10] (SCBT). Image quantification was performed in ImageJ (version 1.53a; freely available at http://imageJ.nih.gov/ij)^90^. Full-size membranes of Western blots from which panels in Figs. 2–5 were prepared are shown in Supplemental Fig. S5–S8.

### Immunofluorescence microscopy

Cells cultured on glass coverslips (Corning) were washed with PBS and fixed using 4% PFA and permeabilized using 0.25% Triton X-100. For imaging after sorting, cells were suspended in PBS at concentrations of ~ 5 × 10^6^ cells/ml and fixed to coverslips by centrifugation. After blocking in 5% BSA, cells were incubated with primary antibodies at 4 °C, washed with PBS, and incubated with highly cross-adsorbed, AlexaFluor-conjugated secondary antibodies at room temperature. Coverslips were mounted to slides using Vectashield hard-set mounting medium with DAPI (Vector Laboratories). Images were acquired using a PerkinElmer UltraVIEW ERS spinning disc confocal imager mounted on a Zeiss AxioVert 200 inverted microscope equipped with a 63×/1.4 Oil DIC Plan-Apochromat 0.19/0.17. Primary antibodies used for fluorescence microscopy were: TFAM (Cell Signaling), cyclin B1 [GNS1] (SCBT), 8-oxo-dG [15A3] (SCBT), COXIV (Cell Signaling), and MAP LC3β [G-9] (SCBT).

### Mitochondrial mass and intracellular ROS measurement

Cells were incubated with Minimum Essential Medium Eagle (MEM) containing 100 nM Mitotracker Red CMXRos for 30 min at 37 °C or 100 nM nonyl acridine orange (Invitrogen) for 15 min. Mitochondrial ROS was measured using 5 µM MitoSOX and cellular ROS using 10 µM dihydroethidium (DHE). Following staining, cells were trypsinized, washed in PBS, and analyzed using a BD FACSCanto II flow cytometer. Propidium iodide was used to exclude dead cells. To assess total cellular levels of ROS, cells were seeded in 96-well plates at a density of 4 × 10^4^ cells, allowed to attach and incubated with 10 µM 2′,7′-dichlorodihydrofluorescein diacetate (DCFA) for 30 min. To assess the effect of the antioxidant N-acetyl cysteine (NAC) cells were treated with 5 mM NAC for 24 h prior to DCFA staining. Fluorescence intensity of DCFA staining was quantified using a microplate reader at an excitation wavelength of 480 nm and an emission wavelength of 530 nm.

For analysis by fluorescence microscopy, cells were fixed with ice-cold methanol (Mitotracker CMXRos) or 4% PFA (NAO staining). Live cells were used for staining with MitoSOX, Mitotracker Deep Red and Lysotracker. Images acquired on a PerkinElmer UltraVIEW ERS spinning disc confocal imager mounted on a Zeiss AxioVert 200 inverted microscope equipped with a 63×/1.4 Oil DIC Plan-Apochromat 0.19/0.17. Mitochondrial morphology was analyzed in Mitotracker-stained cells using the Mito-Morphology Macro^91^ in ImageJ (version 1.53a, freely available at http://imageJ.nih.gov/ij).

### Cellular ATP content

Whole-cell levels of ATP were measured using a luciferase-based assay (ViaLight MDA Plus Detection Kit, Lonza) according to manufacturer’s instructions. Luminescence was quantified in a MicroBeta 2 Scintillation Counter (Perkin Elmer). ADP/ATP ratio was measured using a bioluminescent method based on the conversion of ATP by luciferase. The ADP/ATP Ratio Assay Kit (Abcam) was used according to the manufacturer’s instructions.

### Nocodazole block and release

KSVS1452 (*BLM *^*KO*^), KSVS1454 (*BLM*^*KO/*+^) and wild-type GM00637 (*BLM*^+*/*+^) cells were seeded at ~ 50% confluency in 100 mm culture dishes. Cells were grown in MEM supplemented with 10% FBS and 2 mM thymidine for 24 h to induce G1/S arrest. Cells were washed with PBS, released into MEM supplemented with 10% FBS for 3 h and then incubated for 16 h in MEM containing 1 μM nocodazole (Calbiochem). For release from G2/M arrest, cells were washed with PBS, incubated in MEM supplemented with 10% FBS, collected at indicated time points for 6 h, washed with PBS and either lysed with RIPA buffer for Western blot analysis or prepared for fluorescence activated cell sorting.

### Fluorescence activated cell sorting

To determine DNA content cells were washed twice with sample buffer (0.001% glucose in PBS, filtered through a 0.22 μm filter), resuspended at a concentration of 10^6^ cells/ml, and fixed in 70% ethanol at 4 °C overnight. Fixed cells were stained with propidium iodide (50 µg/ml in sample buffer with 100 Kunitz units/ml RNase A) for 1 h at room temperature. Fluorescence was measured by a BD FACSCantoII flow cytometer and analyzed using BD FACSDiva and FlowJo v. 10.7 software (BD Life Sciences, https://www.flowjo.com/solutions/flowjo/downloads). For cell sorting, asynchronous cells were washed and processed as described above. Fixed cells were sorted into G1 and G2/M populations based on DNA diploidy using a BD FACSMelody cell sorter.

### Measurements of mitochondrial respiration

Mitochondrial respiration was determined in real-time using an XF-96 Extracellular Flux Analyzer (Seahorse Bioscience, Agilent). Cell lines GM00637 (*BLM*^+*/*+^), KSVS1452 (*BLM*^*KO*^) and KSVS1453 (*BLM*^*KO*^) were seeded overnight in Seahorse assay media (Seahorse Bioscience, Agilent), supplemented with 1 mM pyruvate and adjusted to pH 7.4. Oxygen consumption rate (OCR) was measured to establish a baseline of respiratory rate. Subsequently, wells were injected with either 1 µM oligomycin or 0.5 µM carbonyl cyanide p-(trifluoromethoxy) phenylhydrazone (FCCP) to determine respectively ATP turnover (drop in OCR after addition of oligomycin) and reserve respiratory (increase in OCR after addition of FCCP). Finally, 2 µM antimycin A was added to all wells as a control to measure level of non-mitochondrial oxygen consumption. Extracellular acidification rates (ECAR) was determined to establish a measure of glycolytic reserve calculated as change in ECAR due to addition of 1 µM oligomycin. As the different cell lines had different growth rates (Supplemental Fig. S1F) all measures of OCR and ECAR were normalized to basal respiration rate (third measurement, at 22 min).

### Real-time PCR

Total RNA was extracted from cells using the Quick-RNA Miniprep kit (Zymoresearch). Following this, 1 µg of RNA was reverse transcribed using the High-Capacity cDNA Reverse Transcription kit (Thermo Fisher). Real-time PCR was performed using SYBR-Green PCR master mix (Bio-Rad) and an ABI prism 7500 sequence detection system (Applied Biosystems). TFAM and human β-actin primers were designed with Primer3^92^. Values were normalized against β-actin and relative expression was calculated using the delta-delta Ct method.

### DNA fiber assay

DNA replication rates were measured by labeling DNA fibers based on a protocol described earlier^93,94^. Briefly, cells at ~ 50 to 70% confluency were sequentially labeled with 20 μM CldU (Sigma) for 30 min and 100 μM IdU (Sigma) for 30 min. Cells were then resuspended in PBS at a concentration of 1 × 10^6^ cells/ml. Labeled cells diluted with unlabeled cells were allowed to air-dry on a microscopy slide and incubated with 10 μl of lysis buffer (0.5% SDS, 200 mM Tris–HCl, pH 7.4, 50 mM EDTA). Slides were inclined at 15° to stretch the fibers. DNA spreads were fixed by incubation with 3:1 methanol:acetic acid followed by denaturation with 2.5 N HCl for 70 min. Following this, the slides were blocked with 10% goat-serum/PBS-T (PBS+ 0.1% Triton X-100) for 1 h. This was followed by incubation with rat anti-BrdU (anti-CldU; Abcam) and mouse anti-BrdU(anti-IdU; Becton Dickinson) at a dilution of 1:100 and 1:50 respectively in 10% goat-serum/PBS-T(PBS+ 0.1% Triton X-100) for 2 h. Slides were washed with PBS and incubated with AlexaFluor 594 goat anti-rat IgG (H+L) (Invitrogen) and goat anti-mouse (H+L) AlexaFluor 488 Plus (Invitrogen) in the dark for 1 h. Images were acquired using a Keyence BZ-X Fluorescence microscope with a CFI Achromat 60×/0.8 objective.

### Electron microscopy

For transmission electron microscopy (TEM) imaging, BLM-proficient GM00637 and BLM-deficient KSVS1452 human fibroblast cell lines were prepared using high-pressure freezing/freeze substitution fixation (HPF/FS) method. The cells were fixed in 4% paraformaldehyde and 0.5% glutaraldehyde in 0.1 M PBS (pH: 7.4) for 2 h at room temperature and and kept overnight at 4 °C. Cells were washed in 1 × PBS (pH: 7.24) with the aid of a Pelco BioWave Pro laboratory microwave (Ted, Pella, Redding CA, USA). Briefly, the cells were placed into a HPM100 3 mm, 200 type-A aluminum specimen carrier, which was 200 μm in depth and prefilled with a 1-hexadecene cryoprotectant. The cells-containing specimen carrier was covered by the flat side of type-B specimen carrier and high-pressure frozen using HPM 100 (Leica Microsystems, Vienna, Austria). The frozen samples were transferred into LN2 precooled cryo-vials filled with anhydrous acetone containing 1% (w/v) osmium tetroxide and 0.1% uranyl acetate. Freeze substitution (FS) was carried out in a FS unit (AFS2, Leica Microsystems, Vienna, Austria) at − 90 °C for 96 h, followed by two further FS steps at − 45 °C for 24 h and − 20 °C for 24 h. During freeze substitution (FS), the specimen carriers were removed from the frozen samples, and the samples were washed in anhydrous acetone. Finally, the temperature was raised to 4 °C. Resin infiltration steps were carried out with the aid of a laboratory microwave at room temperature. Dehydrated samples were infiltrated in graded acetone-Embed/Araldite epoxy resin with Z6040 embedding primer (Electron Microscopy Sciences, Hatfield, PA, USA) at 30%, 50%, 70%, and 100% and cured at 60 °C. Ultra-thin sections (approximately 120 nm) were collected on Formvar/carbon-coated copper grids and counterstained with 2% aqueous uranyl acetate and Reynold’s lead citrate. Sections were examined with a FEI Tecnai G2 Spirit Twin TEM (FEI Corp., Hillsboro, OR) operated at 120 kV and digital images were acquired with a Gatan UltraScan 2 k × 2 k camera and Digital Micrograph software v. 1.93.1362 (Gatan Inc., Pleasanton, CA, licensed software, https://www.gatan.com/products/tem-analysis/gatan-microscopy-suite-software).

## Supplementary Information

Supplementary Information 1.
