# Supplementary material for: Bloom syndrome DNA helicase deficiency is associated with oxidative stress and mitochondrial network changes
Source: Sci Rep. 2021 Jan 25;11:2157. doi: 10.1038/s41598-021-81075-0 (PMC7835382; doi:10.1038/s41598-021-81075-0)
Supplement: Supplementary file 1 — Supplementary Information 1. [file 41598_2021_81075_MOESM1_ESM.pdf]

Bloom syndrome DNA helicase deficiency is associated with oxidative stress and mitochondrial network changes

by

Veena Subramanian[1*], Brian Rodemoyer[1*], Vivek Shastri[1], Lene J. Rasmussen[2], Claus Desler[2] and Kristina H. Schmidt[1,3#]

[1] Department of Cell Biology, Microbiology and Molecular Biology, University of South Florida, Tampa, FL 33620, USA.

[2] Center for Healthy Aging, Department of Cellular and Molecular Medicine, University of Copenhagen, 2200 Copenhagen, Denmark

[3] Cancer Biology and Evolution Program, H. Lee Moffitt Cancer Center and Research Institute, Tampa, FL 33612, USA.

* authors contributed equally to this work

[#] Correspondence: kschmidt@usf.edu

SUPPLEMENTAL FIGURE LEGENDS

Figure S1. Characterization of the *BLM*^{KO} cell line KSVS1452 generated by CRISPR/Cas9-mediated biallelic disruption of *BLM* exon 8 in GM00637. ***(A)*** Single cell clones were screened by Western blotting for absence of BLM expression. GM08505 is a skin fibroblast cell line from a Bloom's syndrome patient lacking BLM expression. From clones 1, 4, and 6, a fragment encompassing *BLM* exon 8 was amplified by PCR, cloned and sequenced to identify the disruptions in both *BLM* alleles. For exact DNA and amino acid sequence changes identified in clone 6 (KSVS1452) see *Methods*. ***(B)*** Level of sister-chromatid exchange (SCE) is increased in BLM-deficient cells. SCEs were analyzed by phase contrast microscopy of Hoechst/Giemsa-stained metaphase spreads obtained from GM00637 (*BLM*$^{+/+}$) and KSVS1452 (*BLM*^{KO}) cells grown in the presence of BrdU for two rounds of replication as previously described [90]. ***(C)*** Quantification of SCEs in 1500 chromosomes from GM00637 (*BLM*$^{+/+}$), GM08505 (*BLM*$^{-/-}$) and KSVS1452 (*BLM*^{KO}) cells prepared as in *(B)*. Mean ± SD is shown. ***(D)*** Repair of CPT-induced DNA damage is delayed in BLM-deficient cells. GM00637 (*BLM*$^{+/+}$), GM08505 (*BLM*$^{-/-}$) and KSVS1452 (*BLM*^{KO}) cells were exposed to 1 µM CPT for 1 hour to induce replication-dependent DNA breaks. Repair of DNA breaks was assessed by neutral comet assay 24 hours after removal of CPT as previously described [90]. 150 comets from three experiments per time point and cell line were analyzed. Mean ± SD is shown. ***(E)*** BLM-deficient cells are hypersensitive to hydroxyurea. Clonogenic assay to assess survival of GM00637 (*BLM*$^{+/+}$) and KSVS1452 (*BLM*^{KO}) cells after exposure to increasing concentrations of hydroxyurea as previously described [90]. Mean ± SD is shown from three experiments. ***(F)*** Population doubling time (DT) was determined for GM00637 (*BLM*$^{+/+}$), patient-derived GM08505 (*BLM*$^{-/-}$) and KSVS1452 (*BLM*^{KO}) cell lines. SD, standard deviation; hr, hours. ***(G)*** Cell line KSVS1454 (*BLM*$^{KO/+}$) was generated by transfecting KSVS1452 (*BLM*^{KO}) with pcDNA3-BLM expressing wildtype BLM cDNA and selecting stable clones with BLM expression levels similar to that of the BLM-proficient GM00637 cell line. Western blot analysis was performed on whole cell extracts using BLM antibody ab2179 (Abcam). The membrane was cut prior to hybridization between the 75 kDa and 100 kDa bands

of the protein ladder; the upper part of the membrane was incubated with BLM antibody (ab2179, Abcam) and the bottom part with tubulin antibody ([62204] ThermoFisher).

Figure S2. ***(A)*** Cell cycle analysis by flow cytometry of asynchronous cultures of BLM-proficient GM00637 ($BLM^{+/+}$) and KSVS1454 ($BLM^{KO/+}$) cells, and BLM-deficient KSVS1452 (BLM^{KO}) and KSVS1453 (BLM^{KO}) cells. KSVS1454 was generated by stably transfecting KSVS1452 with BLM cDNA. Cells from three individual cultures were analyzed for each cell line. ***(B)*** The fraction of cells in G1, S, and G2/M was calculated using FlowJo v. 10.7 software (BD Life Sciences, https://www.flowjo.com/solutions/flowjo/downloads).

Figure S3. ***(A)*** Confocal microscopy images of fixed GM00637 ($BLM^{+/+}$), KSVS1452 (BLM^{KO}) and GM08505 ($BLM^{-/-}$) cells immuno-stained for endogenous TFAM ([D5C8], Cell Signaling) and counterstained with DAPI. Scale bars, 10 µm. ***(B-C)*** Western blot analysis and quantification showing Nrf1 ([147.1], SCBT) and Nrf2 ([A-10] (SCBT) expression levels from whole cell extracts of GM00637 ($BLM^{+/+}$) and KSVS1452 (BLM^{KO}) cells. Protein extracts from three individual cultures were analyzed for each cell line. Ran ([610340], BD Biosciences) was used as a loading control. Nrf1 and Nrf2 expression levels were quantified using ImageJ v. 1.53 (http://imageJ.nih.gov/ij). Significance of differences was determined by a Student's t-test and is reported as ** $p \leq 0.01$; ns, not significant.

Figure S4. ***(A)*** Cell cycle analysis by flow cytometry of cells blocked with nocodazole (time point 0) and released into drug-free media for 6 hours. KSVS1454 was generated by stably transfecting KSVS1452 with BLM cDNA. ***(B)*** Flow cytometry analysis of sorted G1 and G2/M populations obtained by fluorescence activated cell sorting of asynchronous GM00637 ($BLM^{+/+}$), KSVS1452 (BLM^{KO}), KSVS1453 (BLM^{KO}), and KSVS1454 ($BLM^{KO/+}$) cultures. PI, propidium iodide. Flow cytometry data were analyzed with FlowJo v. 10.7 software (BD Life Sciences, https://www.flowjo.com/solutions/flowjo/downloads).

Figure S5. ***(A)*** Full-size Western blot membrane showing TFAM ([D5C8], Cell Signaling) expression levels in BLM-proficient cell lines GM00637 (*BLM*$^{+/+}$) and in BLM-knockout cell line KSVS1452 (*BLM*^{KO}). GAPDH ([GA1R], Invitrogen) was used as loading control. This Western Blot was used to generate Figure 2F. ***(B)*** Full-size Western blot membrane showing TFAM ([D5C8], Cell Signaling) expression levels in BLM-proficient cell lines GM00637 (*BLM*$^{+/+}$) and in BLM-deficient cell line GM08505 (*BLM*$^{-/-}$). GAPDH ([GA1R], Invitrogen) was used as loading control. This Western Blot was used to generate Figure 2G. ***(C)*** Full-size Western blot membrane showing MAPLC3β ([G-9], SCBT) expression levels in BLM-proficient cell lines GM00637 (*BLM*$^{+/+}$) and in BLM-knockout cell line KSVS1452 (*BLM*^{KO}) following CCCP (10 μM, 6h) and HCQ treatment (30 μg/ml, 6h). Ran ([610340], BD Bioscience) was used as loading control. This Western Blot was used to generate Figure 3A.

Figure S6. ***(A)*** Full-size Western blot membrane showing Mfn1([D-10], SCBT), Opa1([612606], BD Biosciences), and Drp1([6Z-82], SCBT) expression levels in BLM-proficient cell lines GM00637 (*BLM*$^{+/+}$) and KSVS1454 (*BLM*$^{KO/+}$) and in the BLM-knockout cell line KSVS1452 (*BLM*^{KO}). Ran ([610340], BD Biosciences) was used as a loading control. This Western blot was used to generate Figure 4D. Membranes were cut at the 37 kDa band of the protein ladder and the upper part hybridized with Mfn1, Opa1, and Drp1 antibodies and the lower part with Ran antibody. ***(B)*** Full-size Western blot membranes of Drp1 ([6Z-82], SCBT) expression levels and phosphorylation at serine 616 ([D9A1], Cell Signaling) in the GM00637 (*BLM*$^{+/+}$) and KSVS1452 (*BLM*^{KO}) cell lines following G2/M block and release. Ran ([610340], BD Biosciences) was used as a loading control. This Western blot was used to generate Figure 4E. Membranes were cut between the 50 kDa and 37 kDa bands of the protein ladder and the upper part hybridized with Drp1 or Drp1-phospho antibody and the lower part with Ran antibody.

Figure S7. Full-size Western blot membranes showing cyclin B1 and cyclin A expression levels. BLM-proficient GM00637 (*BLM*$^{+/+}$) and KSVS1454 (*BLM*$^{KO/+}$) cells and BLM-deficient KSVS1452 (*BLM*^{KO})

cells were blocked in G2/M with nocodazole, released, and ***(A)*** cyclin B1 ([GNS1], SCBT) and ***(B)*** cyclin A ([BF683], SCBT) levels evaluated for 6 hours. Ran ([610340], BD Biosciences) was used as a loading control. These Western blots were used to generate Figure 5A. All membranes were cut at the 37 kDa band of the protein ladder and the upper part hybridized with cyclin B1 or cyclin A antibody and the lower part with Ran antibody.

Figure S8. ***(A)*** Full-size Western blot membrane showing Nrf1 ([147.1], SCBT) expression levels from whole cell extracts of GM00637 ($BLM^{+/+}$) and KSVS1452 (BLM^{KO}) cells. Three individual cultures were analyzed for each cell line; all six samples were run on the same gel. Ran ([610340], BD Bioscience) was used as a loading control. This Western blot was used to generate Supplemental Fig. S3B. ***(B)*** Western blot membrane showing Nrf2 ([A-10], SCBT) expression levels from whole cell extracts of GM00637 ($BLM^{+/+}$) and KSVS1452 (BLM^{KO}) cells. Three individual cultures were analyzed for each cell line. Ran ([610340], BD Bioscience) was used as a loading control. This Western blot was used to generate Supplemental Fig. S3C. All membranes were cut at the 37 kDa band of the protein ladder and the upper part hybridized with Nrf1 or Nrf2 antibody and the lower part with Ran antibody.

Figure S9. Full-size Western blot membranes used to generate Supplemental Figures ***(A)*** S1A and ***(B)*** S1G. ***(A)*** Screening of BLM ([ab2179], Abcam) expression levels in six candidate clones obtained by CRISPR/Cas9-based disruption of BLM exon 8 in GM00637 ($BLM^{+/+}$) cells. GM00637 ($BLM^{+/+}$) cells and KSVS1452 (BLM^{KO}) cells are included as negative and positive controls, respectively. Tubulin ([62204] ThermoFisher) was used as a loading control. The membrane was cut between the 75 kDa and 100 kDa bands of the protein standard (BioRad) and the upper part of the membrane was hybridized with BLM antibody ([ab2179] Abcam) and the bottom part with tubulin antibody ([62204] ThermoFisher). ***(B)*** BLM expression in three clones obtained by stable transfection of KSVS1452 (BLM^{KO}) cells with wildtype BLM cDNA was analyzed with BLM antibody ab2179 (Abcam). GM00637 ($BLM^{+/+}$) cells were included as a positive control for BLM expression. The membrane was cut between the 75 kDa and 100

kDa bands of the protein standard (BioRad) and the upper part of the membrane was hybridized with BLM antibody ([ab2179] Abcam) and the bottom part with a monoclonal tubulin antibody (Abcam). Proteins were detected with enhanced chemiluminescence (ECL) substrate; exposure times were 30 seconds for the upper part of the membrane incubated with BLM antibody and 10 seconds for the bottom part incubated with tubulin antibody.

Figure S1

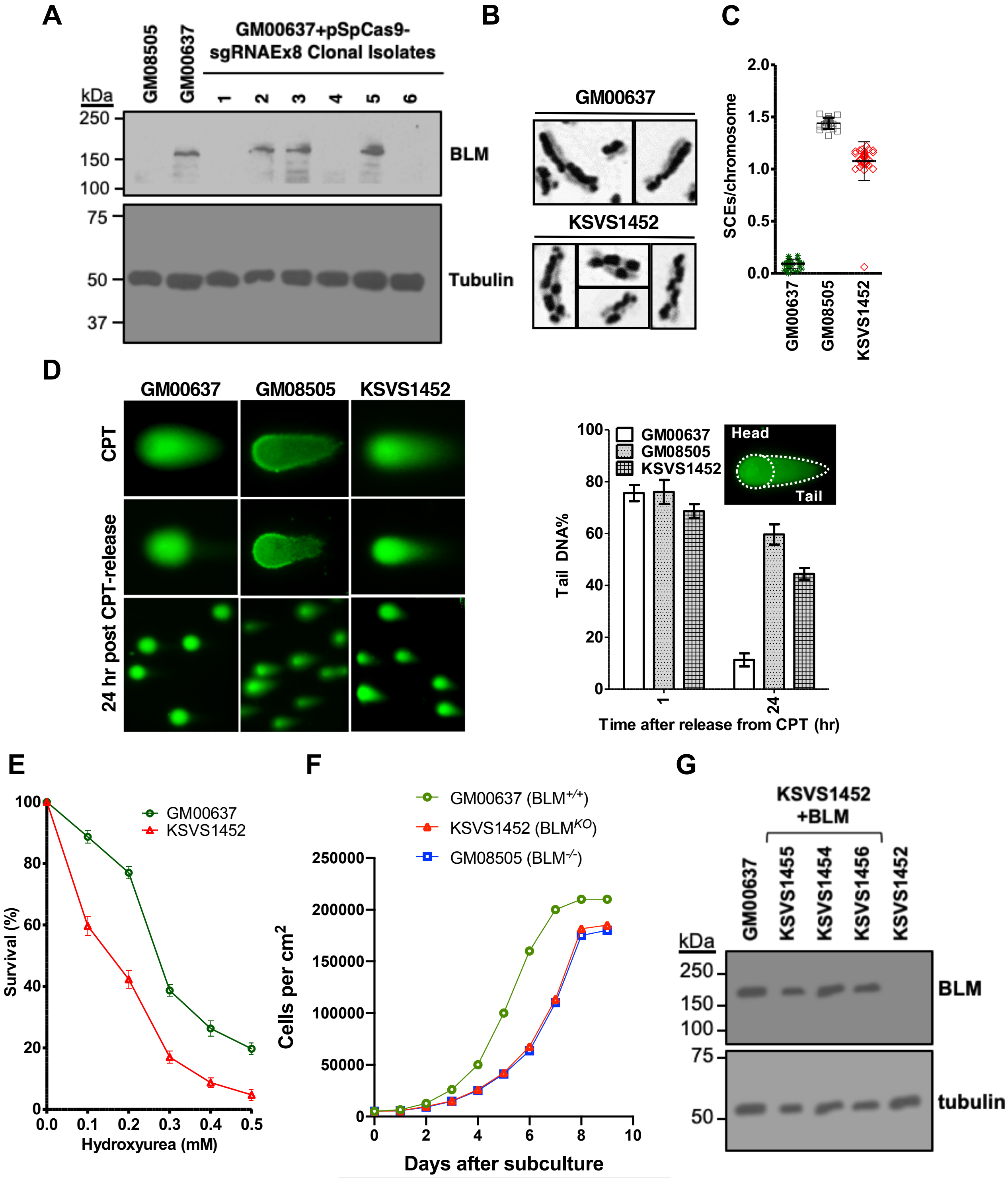

	GM00637	KSVS1452	GM08505
DT (hr)	24.4	34.7	34.2
SD (hr)	0.9	3.5	2.7

Figure S2

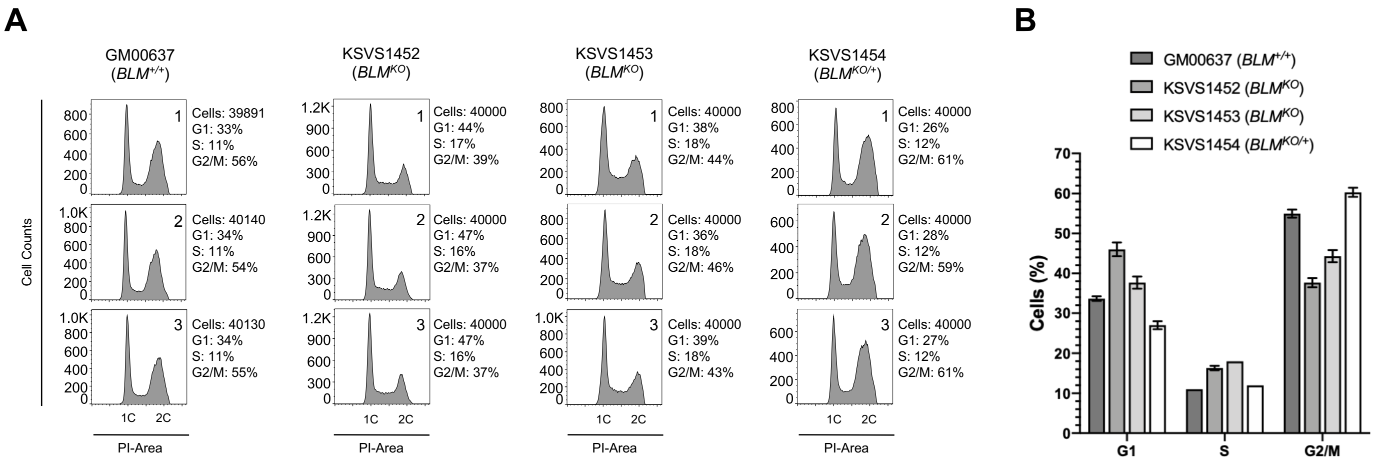
A
GM00637 (BLM+/+)
KSVS1452 (BLMKO)
KSVS1453 (BLMKO)
KSVS1454 (BLMKO/+)
Cell Counts
Cells: 39891 G1: 33% S: 11% G2/M: 56%
Cells: 40140 G1: 34% S: 11% G2/M: 54%
Cells: 40130 G1: 34% S: 11% G2/M: 55%
Cells: 40000 G1: 44% S: 17% G2/M: 39%
Cells: 40000 G1: 47% S: 16% G2/M: 37%
Cells: 40000 G1: 47% S: 16% G2/M: 37%
Cells: 40000 G1: 38% S: 18% G2/M: 44%
Cells: 40000 G1: 36% S: 18% G2/M: 46%
Cells: 40000 G1: 39% S: 18% G2/M: 43%
Cells: 40000 G1: 26% S: 12% G2/M: 61%
Cells: 40000 G1: 28% S: 12% G2/M: 59%
Cells: 40000 G1: 27% S: 12% G2/M: 61%
1C
2C
PI-Area
B
GM00637 (BLM+/+)
KSVS1452 (BLMKO)
KSVS1453 (BLMKO)
KSVS1454 (BLMKO/+)
Cells (%)
G1
S
G2/M

Figure S3

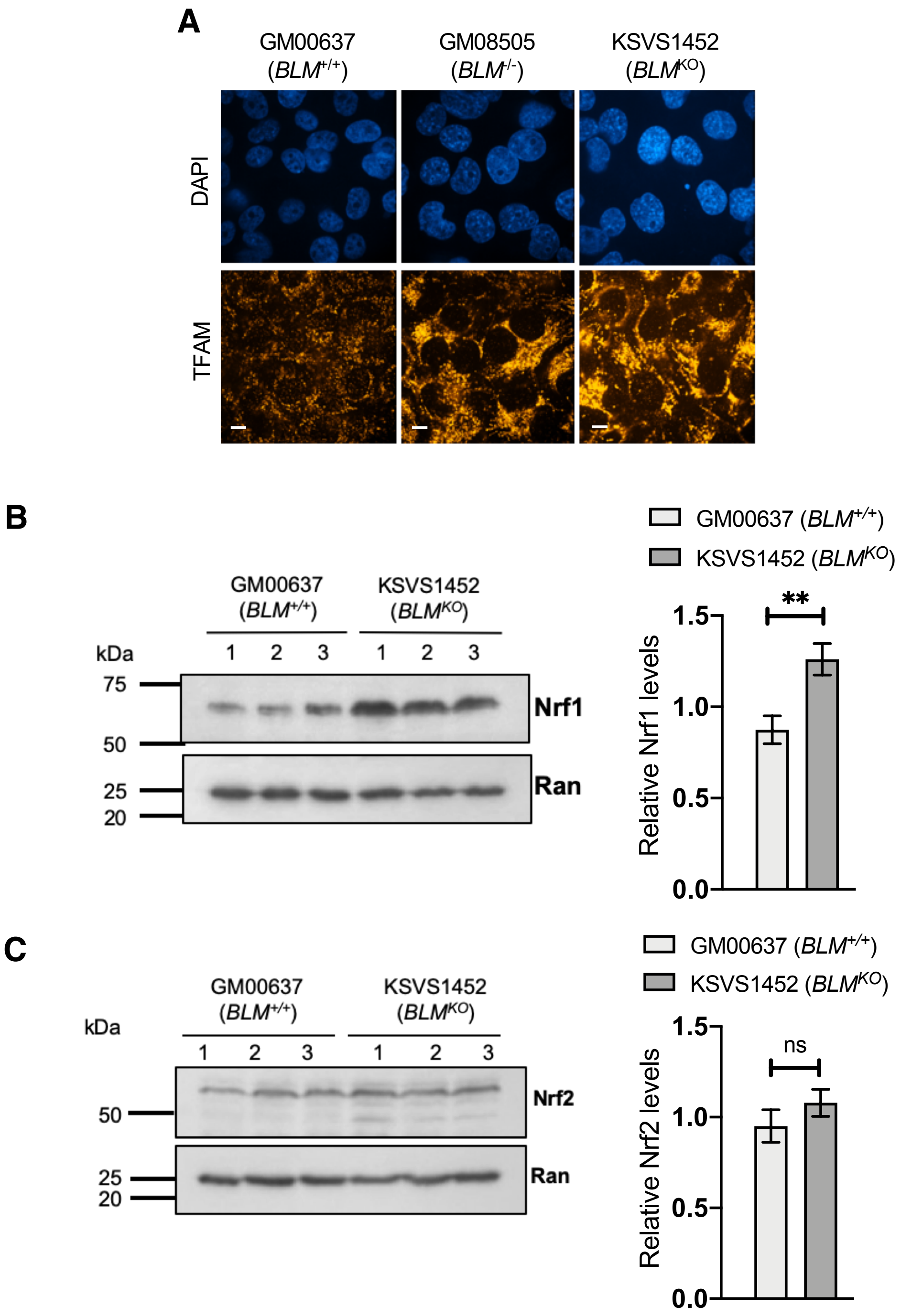

A
GM00637 (BLM+/+)
GM08505 (BLM-/-)
KSVS1452 (BLMKO)
DAPI
TFAM
B
GM00637 (BLM+/+)
KSVS1452 (BLMKO)
kDa
1 2 3 1 2 3
75
50
25
20
Nrf1
Ran
Relative Nrf1 levels
**
1.5
1.0
0.5
0.0
C
GM00637 (BLM+/+)
KSVS1452 (BLMKO)
kDa
1 2 3 1 2 3
50
25
20
Nrf2
Ran
Relative Nrf2 levels
ns
1.5
1.0
0.5
0.0

Figure S4

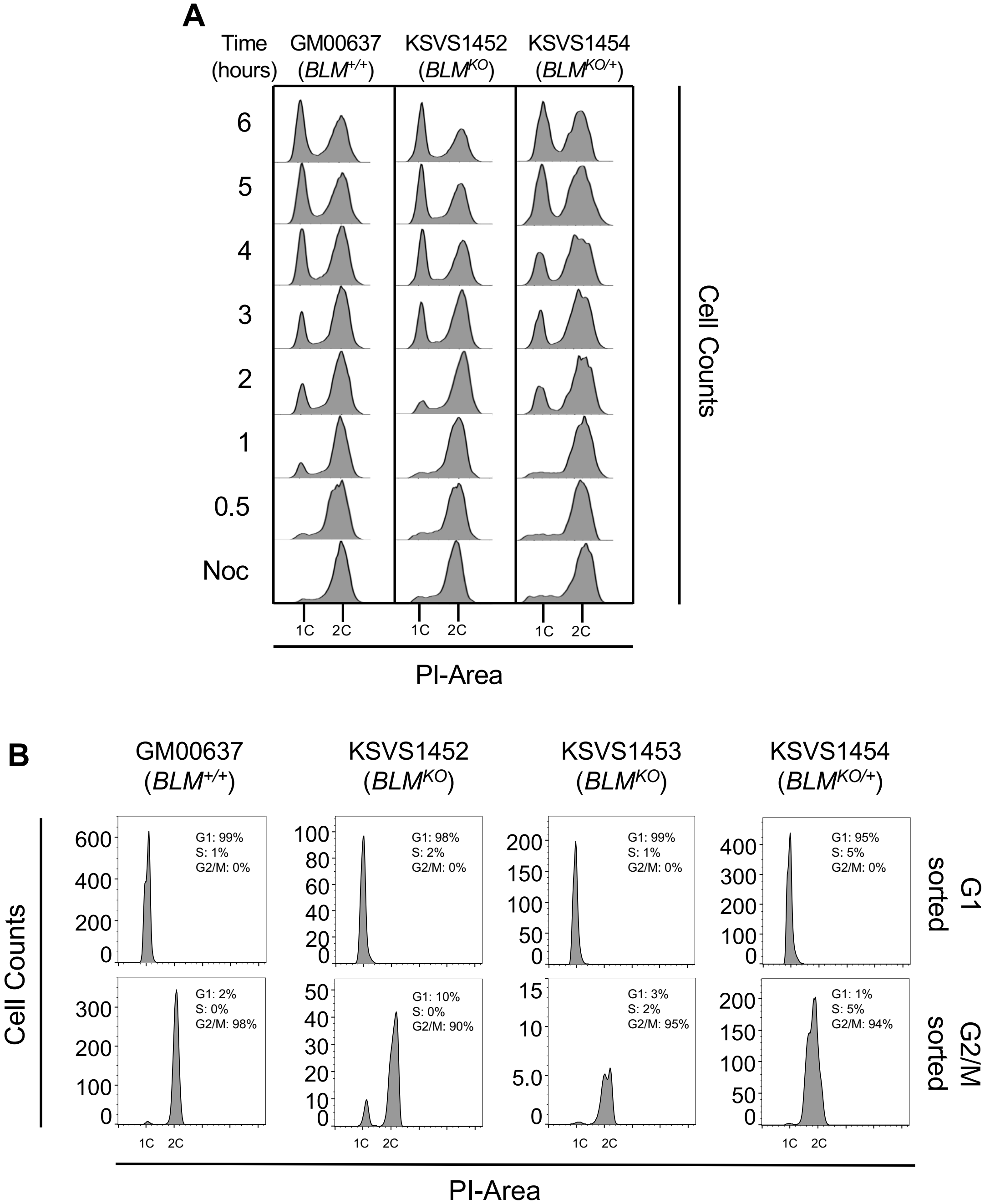
A
Time (hours)
GM00637 (BLM+/+)
KSVS1452 (BLMKO)
KSVS1454 (BLMKO/+)
6
5
4
3
2
1
0.5
Noc
1C 2C
PI-Area
Cell Counts
B
GM00637 (BLM+/+)
KSVS1452 (BLMKO)
KSVS1453 (BLMKO)
KSVS1454 (BLMKO/+)
G1: 99% S: 1% G2/M: 0%
G1: 98% S: 2% G2/M: 0%
G1: 99% S: 1% G2/M: 0%
G1: 95% S: 5% G2/M: 0%
G1: 2% S: 0% G2/M: 98%
G1: 10% S: 0% G2/M: 90%
G1: 3% S: 2% G2/M: 95%
G1: 1% S: 5% G2/M: 94%
G1 sorted
G2/M sorted
Cell Counts
PI-Area

Figure S5

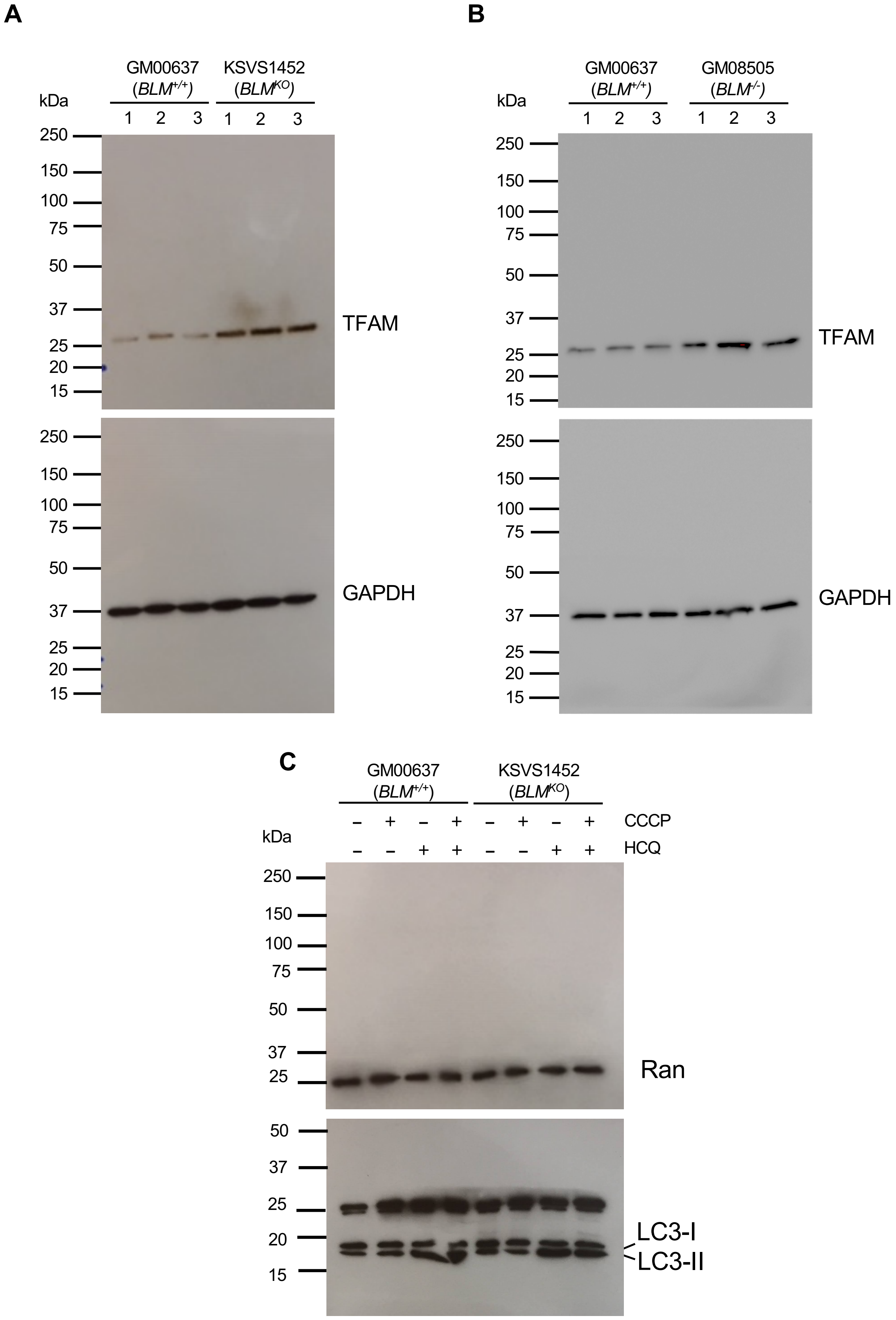
A
GM00637
(BLM+/+)
KSVS1452
(BLMKO)
kDa
1 2 3 1 2 3
250
150
100
75
50
37
25
20
15
TFAM
GAPDH
B
GM00637
(BLM+/+)
GM08505
(BLM-/-)
TFAM
GAPDH
C
GM00637
(BLM+/+)
KSVS1452
(BLMKO)
CCCP
HCQ
Ran
LC3-I
LC3-II

Figure S6

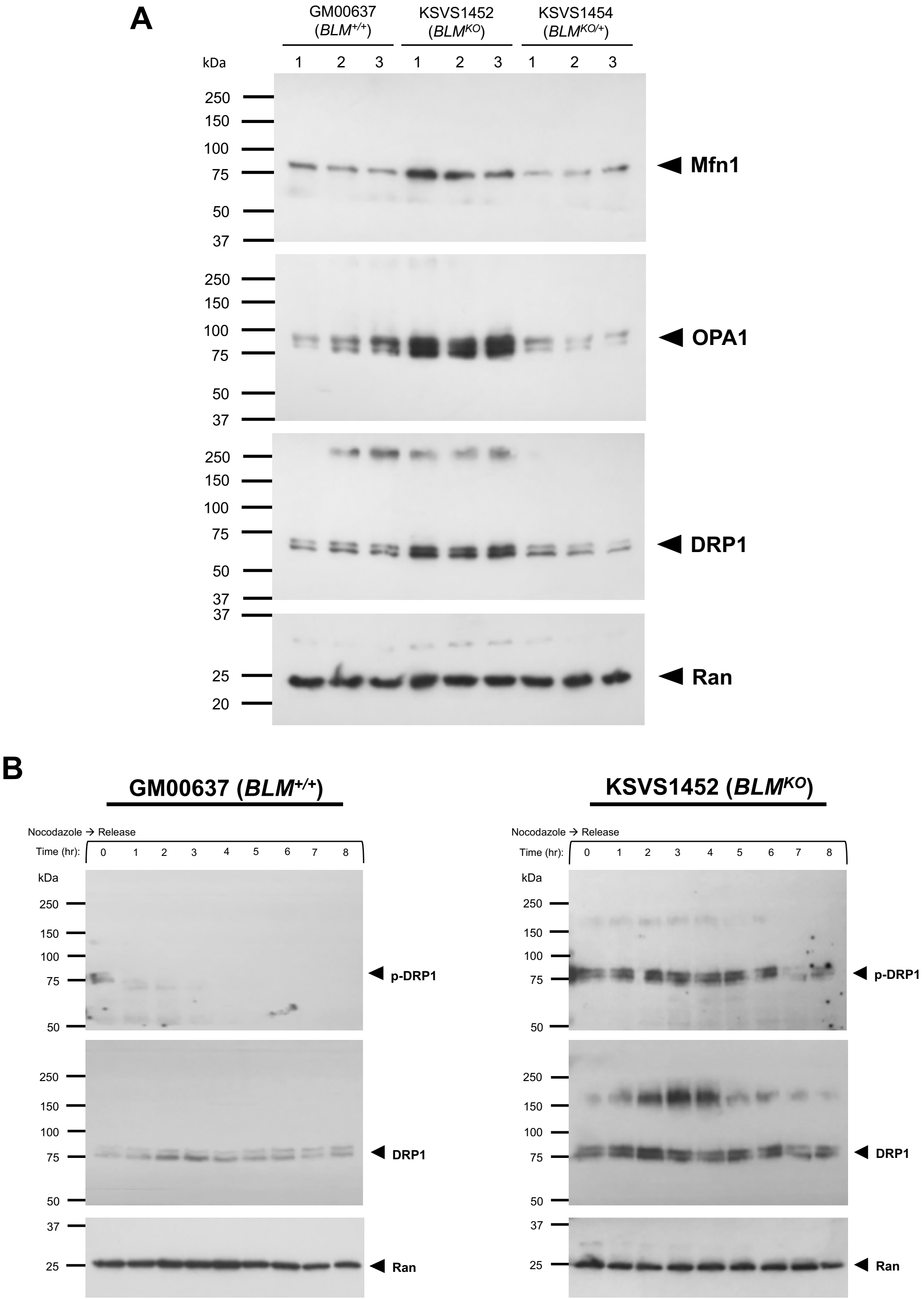
A
GM00637
(BLM+/+)
KSVS1452
(BLMKO)
KSVS1454
(BLMKO/+)
kDa
1 2 3 1 2 3 1 2 3
250
150
100
75
50
37
20
25
Mfn1
OPA1
DRP1
Ran
B
GM00637 (BLM+/+)
KSVS1452 (BLMKO)
Nocodazole → Release
Time (hr):
0 1 2 3 4 5 6 7 8
p-DRP1
DRP1
Ran

Figure S7

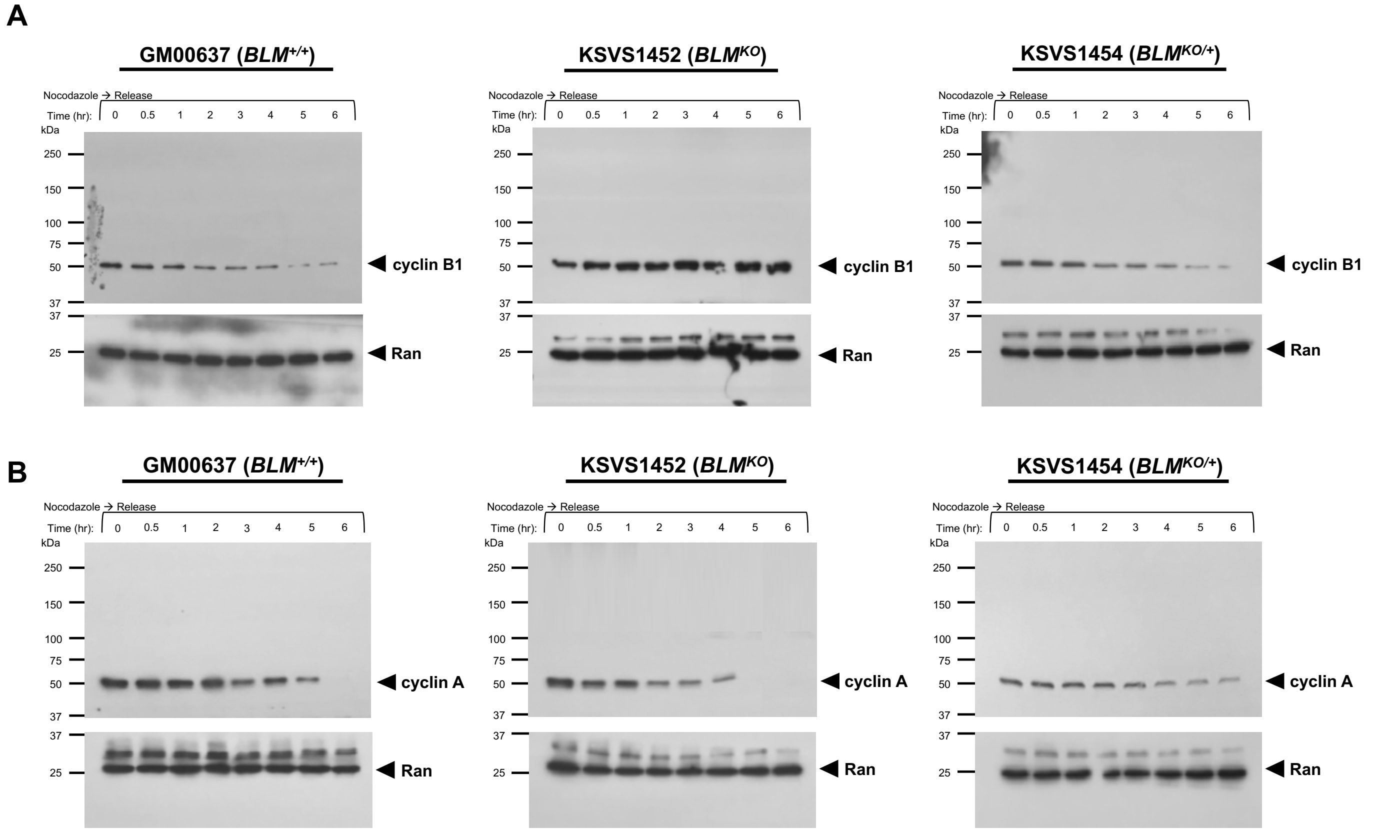
A
GM00637 (BLM+/+)
KSVS1452 (BLMKO)
KSVS1454 (BLMKO/+)
Nocodazole → Release
Time (hr): 0 0.5 1 2 3 4 5 6
kDa
250
150
100
75
50
37
25
cyclin B1
Ran
B
GM00637 (BLM+/+)
KSVS1452 (BLMKO)
KSVS1454 (BLMKO/+)
Nocodazole → Release
Time (hr): 0 0.5 1 2 3 4 5 6
kDa
250
150
100
75
50
37
25
cyclin A
Ran

Figure S8

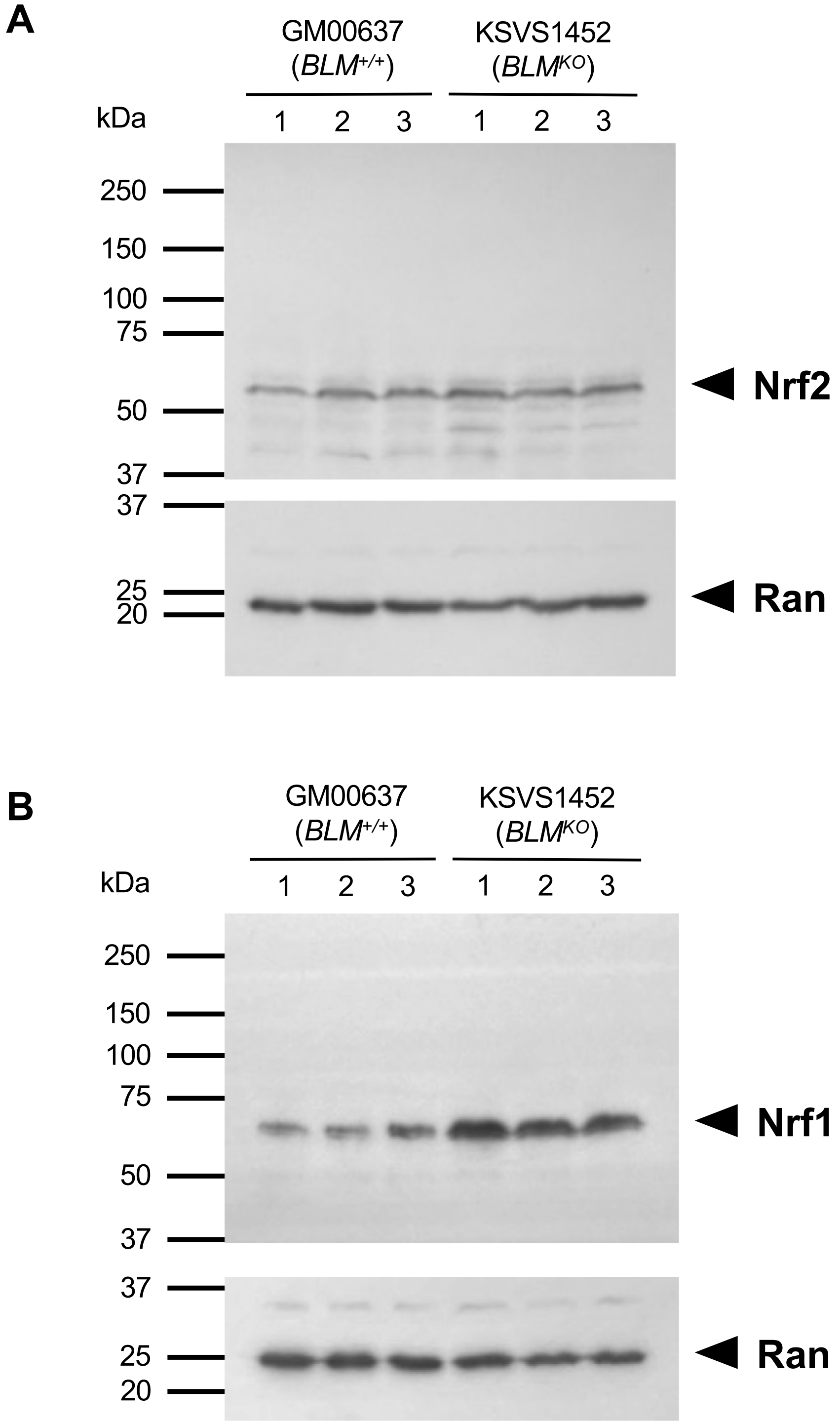
A
GM00637
(BLM+/+)
KSVS1452
(BLMKO)
kDa
1 2 3 1 2 3
250
150
100
75
50
37
Nrf2
37
25
20
Ran
B
GM00637
(BLM+/+)
KSVS1452
(BLMKO)
kDa
1 2 3 1 2 3
250
150
100
75
50
37
Nrf1
37
25
20
Ran

Figure S9

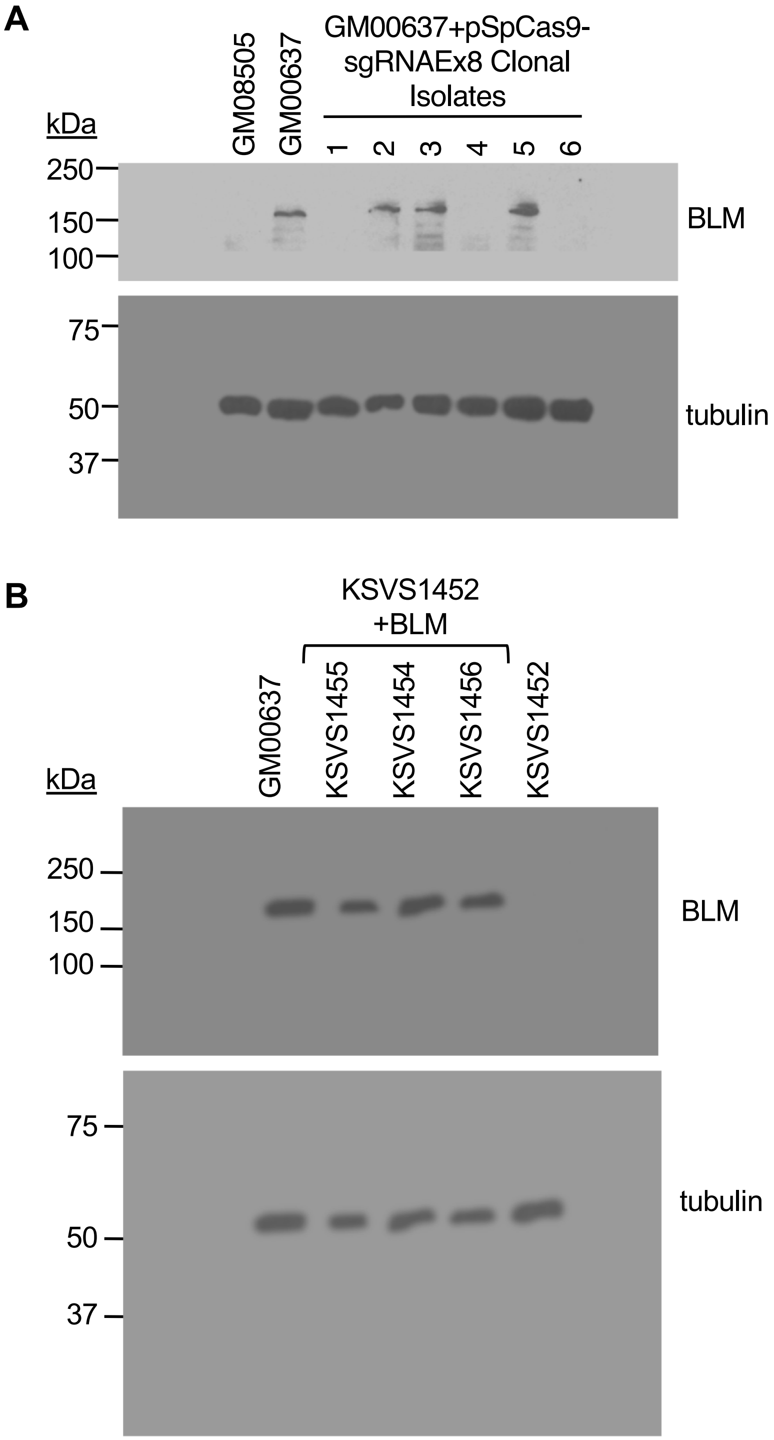
A
GM00637+pSpCas9-sgRNAEx8 Clonal Isolates
kDa
GM08505
GM00637
1
2
3
4
5
6
250
150
100
75
50
37
BLM
tubulin
B
KSVS1452 +BLM
GM00637
KSVS1455
KSVS1454
KSVS1456
KSVS1452
kDa
250
150
100
75
50
37
BLM
tubulin
